# Propofol binds and inhibits skeletal muscle ryanodine receptor 1

**DOI:** 10.1016/j.bja.2024.06.048

**Published:** 2024-09-19

**Authors:** Thomas T. Joseph, Weiming Bu, Omid Haji-Ghassemi, Yu S. Chen, Kellie Woll, Paul D. Allen, Grace Brannigan, Filip van Petegem, Roderic G. Eckenhoff

**Affiliations:** 1 Department of Anesthesiology and Critical Care, Perelman School of Medicine, University of Pennsylvania, Philadelphia, PA, USA; 2 Department of Biochemistry, University of British Columbia, Vancouver, BC, Canada; 3 Department of Anesthesiology, University of Tennessee, Knoxville, TN, USA; 4 Department of Physics and Center for Computational and Integrative Biology, Rutgers University, Camden, NJ, USA

**Keywords:** free energy perturbation, malignant hyperthermia, photoaffinity labelling, propofol, ryanodine receptor 1, skeletal muscle

## Abstract

**Background:**

As the primary Ca^2+^ release channel in skeletal muscle sarcoplasmic reticulum (SR), mutations in type 1 ryanodine receptor (RyR1) or its binding partners underlie a constellation of muscle disorders, including malignant hyperthermia (MH). In patients with MH mutations, triggering agents including halogenated volatile anaesthetics bias RyR1 to an open state resulting in uncontrolled Ca^2+^ release, increased sarcomere tension, and heat production. Propofol does not trigger MH and is commonly used for patients at risk of MH. The atomic-level interactions of any anaesthetic with RyR1 are unknown.

**Methods:**

RyR1 opening was measured by [^3^H]ryanodine binding in heavy SR vesicles (wild type) and single-channel recordings of MH mutant R615C RyR1 in planar lipid bilayers, each exposed to propofol or the photoaffinity ligand analogue *m*-azipropofol (AziP*m*). Activator-mediated wild-type RyR1 opening as a function of propofol concentration was measured by Fura-2 Ca^2+^ imaging of human skeletal myotubes. AziP*m* binding sites, reflecting propofol binding, were identified on RyR1 using photoaffinity labelling. Propofol binding affinity to a photoadducted site was predicted using molecular dynamics (MD) simulation.

**Results:**

Both propofol and AziP*m* decreased RyR1 opening in planar lipid bilayers (*P*<0.01) and heavy SR vesicles, and inhibited activator-induced Ca^2+^ release from human skeletal myotube SR. Several putative propofol binding sites on RyR1 were photoadducted by AziP*m*. MD simulation predicted propofol *K*_D_ values of 55.8 μM and 1.4 μM in the V4828 pocket in open and closed RyR1, respectively.

**Conclusions:**

Propofol demonstrated direct binding and inhibition of RyR1 at clinically plausible concentrations, consistent with the hypothesis that propofol partially mitigates malignant hyperthermia by inhibition of induced Ca^2+^ flux through RyR1.

**Editor's key points**

• Type 1 ryanodine receptor (RyR1) is the primary Ca^2+^ release channel in skeletal muscle sarcoplasmic reticulum, and mutations underlie various muscle disorders, including malignant hyperthermia, which is triggered by channel opening by volatile anaesthetics, but not propofol.
• Propofol and the photoaffinity ligand analogue *m*-azipropofol (AziP*m*) decreased RyR1 opening and inhibited activator-induced Ca^2+^ release from human skeletal myotube sacroplasmic reticulum.
• Several putative propofol binding sites on RyR1 were identified by photolabeling with by AziP*m* and molecular dynamic simulation.
• Propofol bound to and inhibited RyR1 at clinically plausible concentrations, suggesting that propofol might partially mitigate malignant hyperthermia by inhibition of induced Ca^2+^ flux through RyR1.

Ryanodine receptor 1 (RyR1) is the primary Ca^2+^ release channel in the sarcoplasmic reticulum (SR) of skeletal muscle. It is a critical element of excitation-contraction coupling, along with voltage-gated calcium channel Ca_V_1.1, STAC3, and Junctophilin-1 or Junctophilin-2.^1^ A complex interplay of allosteric mechanisms controls the opening of RyR1, including small molecules, protein binding partners, and post-translational modifications.^2^

Dysregulation of RyR1 underlies the pathophysiology of a constellation of muscle disorders, including central core disease,^3^ multiminicore disease,^4^ and malignant hyperthermia (MH).^5^ During an MH episode, RyR1 is biased to an open state, resulting in uncontrolled flow of Ca^2+^ ions out of the SR, causing heat production and often muscle rigour. Returning this excess of Ca^2+^ to the SR consumes ATP, creating a hypermetabolic state associated with acidosis, hyperkalaemia, rhabdomyolysis, and hyperthermia. The necessary conditions for MH include one of many causative mutations in RyR1, Ca_V_1.1, STAC3, or an unidentified additional gene together with a triggering drug (volatile anaesthetics or suxamethonium). Even with all factors, an MH episode might not trigger. It is presumed, but not directly shown, that triggering drugs bind to RyR1 or its partners.

Anaesthesia must often be continued during an MH episode to safely conclude the surgical procedure. Nontriggering agents are substituted for triggering agents. Propofol, a γ-aminobutyric acid type A (GABA_A_) receptor agonist, is the general anaesthetic of choice because it has not been reported to trigger MH,^6^ but it does have many similar targets and effects as volatile anaesthetics.^7^ Thus it would be surprising if propofol had no effect on RyR1, at which the volatile anaesthetics have a strong effect. Here, we provide evidence that propofol binds directly to RyR1 and inhibits its opening at clinically reasonable concentrations. We also identify binding sites and computationally show that propofol binding affinity makes them plausible RyR1 inhibitory sites.

## Methods

Detailed methods are available in the Supplementary material.

### [^3^H]Ryanodine binding assay

This was conducted as described.^8^ Heavy SR (HSR) vesicles were provided by Francisco Alvarado (Cardiovascular Research Center, School of Medicine and Public Health, University of Wisconsin, Madison, WI, USA). Propofol (1–100 μM) or AziP*m* (1–48 μM) was added to HSR protein in 200 mM KCl, 100 mM 4-(2-hydroxyethyl)-1-piperazineethanesulfonic acid (HEPES) buffer (pH 7.2), 5 nM [^3^H]ryanodine (56 Ci mmol^−1^), 1 mM EGTA, and enough CaCl_2_ to set free [Ca^2+^] at 100 nM (pCa 7) or 10 μM (pCa 5). Inclusion of 20 μM unlabelled ryanodine in some samples allowed for nonspecific binding estimations. After incubation for 2 h at 37°C, filters were washed and [^3^H]ryanodine determined with liquid scintillation counting. Experiments were done in triplicate.

### Purification of rabbit or pig RyR1

Frozen rabbit or pig skeletal muscle (∼200 g) was blended, centrifuged, filtered, and centrifuged again at higher speed at 4°C. Pellets were solubilised in calcium-free buffer containing 1% 3-[(3-cholamidopropyl)dimethylammonio]-1-propanesulfonate hydrate (CHAPS), and 0.2% soybean phosphatidylcholine with 100 μl of protease inhibitor cocktail. His-GST-FKBP12.6 (∼5 mg, made in-house) was added, followed by ultracentrifugation. The supernatant was incubated at 4°C with pre-equilibrated GS4B resin (Cytiva, Marlborough, MA, USA). RyR1 was eluted from the resin using TEV protease (made in-house). The eluents were further concentrated and purified with gel filtration using Superose 6 10/300 GL (Cytiva, Marlborough, MA, USA). Fractions containing RyR1 complexes measured by absorbance at 280 nm and were concentrated to ∼2 mg ml^-1^.

### RyR1 proteolipsome reconstitution

RyR1 was reconstituted into proteoliposomes as described.^9^ Briefly, a 5:3 mixture of 1,2-dioleoyl-*sn*-glycero-3-phosphoethanolamine (DOPE) and 1,2-dioleoyl-*sn*-glycero-3-phosphocholine (DOPC) (Avanti Polar Lipids, Alabaster, AL, USA) were dried into a thin film and solubilised with 400 μl of rabbit RyR1 (0.7 mg ml^−1^). Following dialysis, the samples were aliquoted, flash-frozen in liquid nitrogen, and stored at −80°C.

### Planar lipid bilayer methods: single channel recordings

Using the Orbit mini setup and EDR2 software (Nanion Technologies, Livingston, NJ, USA), recordings were obtained in parallel with multielectrode-cavity-array chips (Ionera Technologies, Freiburg, Germany). The *cis* and *trans* chambers contained symmetrical solutions of 250 mM HEPES, 150 mM KCl, 1 mM EGTA (pH 7.3), 0.2 mM CaCl_2_ ([Ca^2+^] free = 0.1 μM). To promote fusion to prepared suspended bilayers, 5% glycerol was incorporated into the proteoliposomes and 1–2 μl RyR1 proteoliposomes were added to the *cis* chamber. To further promote fusion, voltage was maintained at +40 mV. Recordings were started at the point of successful RyR1 insertion. Propofol was introduced to the *cis* chamber and concentrations determined by absorbance at 270 nm.^10^ All RyR1 measurements were conducted at 22°C and constant voltage of –60 mV. Recordings were filtered at final bandwidth of 10 kHz. Clampfit software (10.6, Molecular Devices, San Jose, CA, USA) was used to analyse current traces and only channels with a conductance > 700 pS were included in the analysis.^11^

### Calcium imaging in human skeletal myotubes

Human skeletal muscle cells were maintained in growth medium in a 5% CO_2_ atmosphere at 37°C. After passage and to induce differentiation, plated cells were incubated overnight in growth medium, then in differentiation medium, the latter changed every other day. Multinuclear myotubes typically formed within 5–6 days. Differentiated myotubes were loaded with 1.5 μM Fura-2 AM marker in 20% bovine serum albumin (BSA) and incubated for 15 min to allow de-esterification. Fura-2 binds to intracellular Ca^2+^, with the ratio of emission at 340 nm and 380 nm directly related to the concentration of Ca^2+^.^12^, ^13^, ^14^ Emission ratio was measured using a fluorescent microscope with a cooled high-speed digital video camera and MetaFluor software (version 7.10.4.407, MetaMorph 2020, Molecular Devices, LLC, San Jose, CA, USA). Changes in Fura-2 fluorescence were measured for each drug concentration (*n*=40–50 cells): ryanodine (2–1000 nM) or propofol (2–300 μM). Data were normalised to the maximal response of cells, and IC_50_ calculated by fitting to Hill curves using PRISM 10 software (GraphPad Software, San Diego, CA, USA).

### Photolabelling of RyR1-FKBP12.6

AziP*m* (5 μM) was added (with and without 200 μM propofol to determine specificity) to purified RyR1-FKBP12.6 at a final protein concentration of 1 μg μl^−1^. Samples were equilibrated on ice in the dark for 5 min then irradiated for 30 min at 350 nm with an RPR-3000 Rayonet lamp in 1-mm path length quartz cuvettes through a 295-nm glass filter (Newport Corporation, Franklin, MA, USA).

### In-solution protein digestion

After UV exposure, proteins were precipitated in acetone, pelleted, washed, and air-dried before resuspension in 50 mM Tris–HCl, pH 8.0, 1% Triton X-100, and 0.5% SDS. Insoluble debris was pelleted and resuspended in NH_4_HCO_3_. Samples were treated with dithiothreitol (DTT) and iodoacetamide (IAA) before sequencing-grade modified trypsin was added at a 1:20 protease/protein ratio (w/w) with additional of 0.2% (w/v%) ProteaseMAX (Promega, Madison, WI, USA) surfactant. Proteins were digested and then diluted with NH_4_HCO_3_ and 0.02% ProteaseMAX surfactant before the addition of sequencing-grade chymotrypsin at 1:20 protease/protein ratio (w/w). Proteins were digested and acidified before centrifugation to remove insoluble debris. Finally, the sample was desalted using C18 stage tips, dried under vacuum and resuspended in 0.1% formic acid before mass spectrometry.

### In-gel protein digestion

Photolabelled proteins were separated by SDS-PAGE; the rRyR1 band was excised, destained, dehydrated, and dried before proteins were reduced by 5 mM DTT and 50 mM NH_4_HCO_3_. Samples were then alkylated with 55 mM IAA in 50 mM NH_4_HCO_3_, dehydrated, and dried before resuspension in 0.2% ProteaseMAX surfactant and 50 mM NH_4_HCO_3_. After this point the digestion, suspension, and extraction were essentially identical to the above in-solution scheme.

### Mass spectrometry

Mass spectrometry was performed as reported.^15^ Briefly, desalted peptides were injected into a Thermo LTQ Orbitrap XL Mass Spectrometer (Thermo Fisher Scientific, Waltham, MA, USA) or an Orbitrap Elite Hybrid Ion Trap mass spectrometer. Peptides were eluted with 100 min with linear gradients of acetonitrile in 0.1% formic acid. Spectral analysis was conducted using MaxQuant^16^ to search b and y ions against the rRyR1 sequence. All analyses included dynamic oxidation of methionine (+15.9949 m/z) as well as static alkylation of cysteine (+57.0215 m/z; iodoacetamide alkylation). Photolabelled peptides were searched for the additional dynamic AziP*m* modifications. Both in-solution and in-gel sequential trypsin/chymotrypsin digests were searched without enzyme specification with a false discovery rate of 0.01. Samples were analysed in triplicate and samples containing no photoaffinity ligand (controls) were run to identify false positives.

### Molecular dynamics simulations

We used cryo-electron microscopy models (PDB: 6X34, open state; and 6X36, closed-state) of pig R615C RyR1.^9^ Only the central pore domain of RyR1, itself a functional channel,^17^ was simulated, as the entire protein is computationally impractical for demanding free energy perturbation (FEP) molecular dynamics (MD) simulations. Systems were constructed using CHARMM-GUI.^18^ All simulations were conducted using NAMD 2.14 or 3^19^ with CHARMM36 force field, existing propofol parameters^20^, ^21^, ^22^ and TIP3P water. Production simulations were conducted in the isothermic–isobaric ensemble with Langevin thermostat. The lipid bilayers consisted of 70% 1-palmitoyl-2-oleoyl-*sn*-glycero-3-phosphocholine (POPC) and 30% cholesterol.

Streamlined Alchemical Free Energy Perturbation (SAFEP) methodology^23^^,^^24^ was used to determine the absolute binding free energy of propofol $\left({\Delta G}_{\text{bind}}^{\circ }\right)$. SAFEP uses a limited set of restraints on the ligand to maintain its bound conformation during alchemical transformations and improve sampling of states that most contribute to ${\Delta G}_{\text{bind}}^{\circ }$. The restraints are then corrected to yield an accurate absolute ${\Delta G}_{\text{bind}}^{\circ }$. The overall expression is:

$${\Delta G}_{\text{bind}}^{\circ }={–\Delta G}_{\text{site}}^{*}+{\Delta G}_{\text{DBC}}-{\Delta G}_{V}^{\circ }+{\Delta G}_{\text{bulk}}^{*}$$

where ${\Delta G}_{\text{bulk}}^{*}$ is the energy of decoupling the unbound ligand from solvated to gas phase, ${\Delta G}_{V}^{\circ }$ and ${\Delta G}_{\text{DBC}}$ energies of volumetric and distance-from-bound-conformation (DBC) restraints, respectively, and ${–\Delta G}_{\text{site}}^{*}$ is the energy of coupling the ligand from gas phase to the protein-bound state. ${\Delta G}_{\text{bulk}}^{*}$ and ${\Delta G}_{\text{site}}^{*}$ were calculated using FEP MD, ${\Delta G}_{\text{DBC}}$ using thermodynamic integration, and ${\Delta G}_{V}^{\circ }$ parametrically. The Bennett acceptance ratio method was used to calculate free energy differences in FEP calculations.

## Results

### *m*-Azipropofol and propofol decrease the proportion of open wild-type RyR1

**Fig 1:**
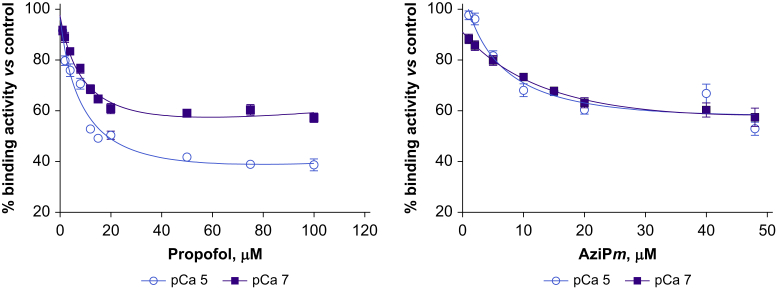
[^3^H]Ryanodine binding to skeletal muscle heavy sarcoplasmic reticulum vesicles as a function of propofol (left) or AziP*m* concentration (right). Values expressed as percentage of control (0 μM propofol or AziP*m*).

We measured [^3^H]ryanodine binding, reflecting the proportion of open RyR1 in HSR vesicles, as a function of *m*-azipropofol (AziP*m*) and propofol concentrations.^8^ At low [Ca^2+^] (pCa 7, 100 nM), AziP*m* and propofol each reduced the proportion of open channels, with IC_50_ values of 6.9 μM and 5.8 μM, respectively. At activating [Ca^2+^] (pCa 5, 10 μM), a similar trend was observed, with IC_50_ values of 4.8 μM and 6.7 μM, respectively (Fig. 1). With AziP*m*, pCa 7 was a sufficient activating concentration to observe its inhibitory effects.

### AziP*m* and propofol inhibit R615C RyR1 channel opening in planar lipid bilayers

**Fig 2:**
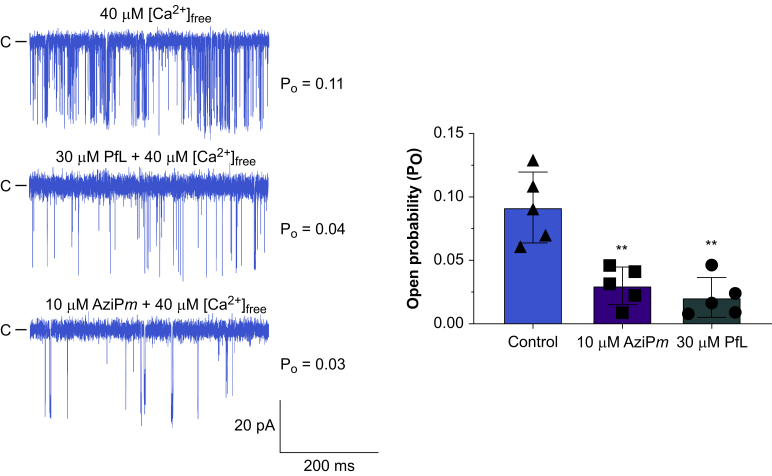
Ryanodine receptor 1 channel opening probability as a function of propofol and AziP*m* concentration in phospholipid bilayers. ∗∗*P*<0.01 *vs* control, Mann-Whitney test (*n*=5).

The R615C mutation predisposes pigs to MH-like porcine stress syndrome,^25^ serving as a model of human MH; the analogous human mutation is R614C. We measured the channel open probability of homozygous pig R615C RyR1 reconstituted in planar lipid bilayers (PLBs) as a function of AziP*m* and propofol concentrations with an activating concentration of Ca^2+^ (40 μM). Without drug, channel open probability was 0.11. This decreased, respectively, to 0.03, 0.07, and 0.04 with 10 μM AziPm, 10 μM propofol, and 30 μM propofol (Fig. 2).

### Propofol inhibits activator-mediated RyR1 channel opening in wild-type cultured human skeletal myotubes

**Fig 3:**
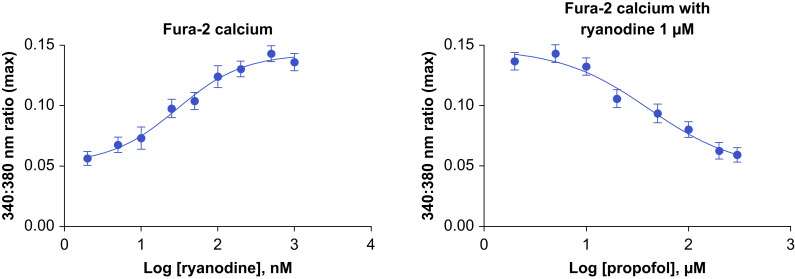
A 340:380 nm ratio with activators and ryanodine as a function of propofol concentration. Greater open probability with higher ryanodine concentration is implied (left). At a constant concentration of ryanodine, increasing propofol concentration results in lower open probability.

We studied the effect of propofol on intracellular Ca^2+^ concentration in wild-type human skeletal muscle myotubes (HSM) in the presence of the RyR1 activator ryanodine. Higher 340:380 nm ratios measured using Fura-2 Ca^2+^ imaging indicate increased Ca^2+^ release attributable to RyR1 opening. Without propofol, the 340:380 nm ratio saturated at ∼ 1 μM ryanodine (Fig. 3), indicating that ryanodine at 1 μM is maximally potent. When propofol was added to HSM Fura-2 preparations containing 1 μM ryanodine, the 340:380 nm ratio decreased as propofol concentration increased, suggesting propofol inhibits RyR1 opening (mean IC_50_ = 29.5 μM [se=0.1 μM]).

### AziP*m* binding sites identified on RyR1 by photoaffinity labelling

**Fig 4:**
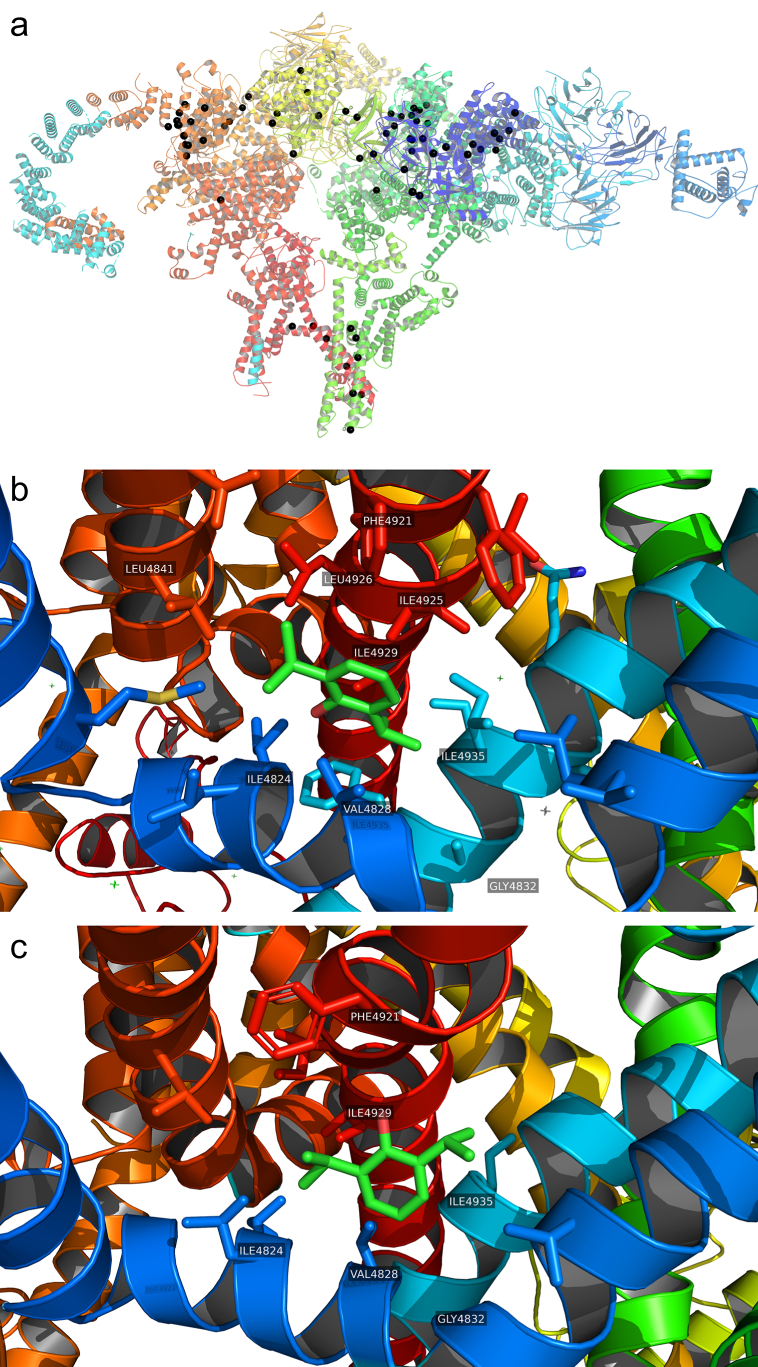
(a) Ryanodine receptor 1 (RyR1) residues photoadducted by AziP*m*, shown as black spheres. For visual clarity only two of the four monomers are shown. (b) V4828 site in open-state RyR1 occupied by propofol. (c) V4828 site in closed-state RyR1 occupied by propofol. Representative poses taken from equilibrium molecular dynamics simulations.

AziP*m* is chemically and functionally similar to propofol,^26^ so photoadducted residues likely indicate propofol binding sites. Photoaffinity labelling was conducted with RyR1 purified from skeletal muscle of wild-type rabbit (*Oryctolagus cuniculus*), wild-type pig (*Sus scrofa*), and R615C pig. Rabbit and pig RyR1 have 97% sequence identity with human RyR1; most nonidentical residues reside outside the transmembrane domain. A preparation of RyR1 and FKBP12.6 (calstabin-2, which stabilises the closed state likely favoured by propofol and AziP*m*) was incubated with AziP*m* and irradiated.^26^ Photoadducted RyR1 residues were identified using mass spectrometry. Sequence coverage was 83%, 87.5%, and 80.2% across rabbit, pig WT, and pig R615C proteins, respectively. Coverage maps and spectra are in Supplementary Figures S2–S7. No photolabelled peptides were identified in nonphotolabelled control samples. Pretreatment with 200 μM propofol prevented adduction by AziP*m* in most sites (Supplementary Tables S1 - S3) implying AziP*m* and propofol binding sites are similar. Taken together with the PLB data, these data suggest that AziP*m* and propofol bind and act in the same locations on RyR1. The identified sites are listed in Table 1 and depicted in Figure 4. Unless otherwise noted, we refer to all photoadducted sites by their sequence locations in the rabbit RyR1 unless present only in pig RyR1. Many of the sites are adjacent to functionally significant RyR1 regions.^27^

**Table 1:** AziP*m* photoadducted sites in ryanodine receptor 1. WT, wild type.

Domain	Rabbit	Pig WT	Pig R615C	Notes
Cytoplasmic		V1689	V1689	Junctional solenoid (JSol)
	T2069	L2068	R2072	JSol
		I2183	I2183	Bridging solenoid (BSol)
	M2440			BSol
	C2555			BSol
Core solenoid	M3638	M3634	M3634	BSol
	L3798	L3793	L3793	Central solenoid (CSol)
	I4058	I4053	I4053	CSol
	D4220	I4213	I4213	Thumb-and-forefinger (TaF), near ATP binding site
Transmembrane	Y4554	L4553	L4553	Pseudo voltage sensing domain (pVSD)
	F4568	A4572	A4572	S1
	Y4715	Y4713	Y4713	S2–S3 linker; near caffeine site
	I4737	I4735	I4735	S2–S3 linker; caffeine site
	L4827	V4828	I4824	S4–S5 linker
			V4828	
	L4850	L4848	L4848	S5
		L4909	L4909	Pore

### Propofol binds near V4828

The photoadducted V4828 residue in the S4–S5 linker forms part of a binding pocket surrounded by lipophilic α-helices, and is adjacent to the pore lumen (Fig. 1). Mutations in this region (T4825I, H4832Y in rabbit) increase Ca^2+^ affinities for activation and decrease it for deactivation *in vitro*: channel open probabilities are increased at both low and high [Ca^2+^], but not at intermediate concentrations,^27^ with predicted affinity similar to clinical concentration. The specific photoadducted atom (e.g. side chain or carbonyl) is by definition not identified. As neither the bound pose of the nonphotolysed parent ligand nor its affinity are determined experimentally, we used MD simulation to predict these. Because it would be computationally prohibitive to simulate the entire RyR1, we included only the pore-containing transmembrane domain (TMD, residues 4546–5029), itself a functional channel,^17^ embedded in a lipid bilayer surrounded by 0.15 M KCl. We initialised each system by manually placing a single propofol molecule in one V4828 pocket followed by minimisation and equilibration MD simulation. This approach imposes no energetic penalty while the ligand is in the pocket, allowing it to locate a local energetic minimum.^28^

In 50 ns production MD simulations, propofol remained in a stable orientation (Fig. 4). In both open- and closed-state RyR1, its hydroxyl group remained oriented away from V4828, although with different orientations. We used SAFEP MD^23^ to predict its binding affinity, with aqueous propofol as the unbound reference point. The predicted Gibbs free energy of propofol binding to open-state RyR1 was ${\Delta G}_{\text{bind}}^{\circ }$ = –5.9 [se 0.1 kcal mol^−1^], corresponding to *K*_D_ = 55.8 μM (95% confidence interval [CI]: 40.3–77.3 μM). This includes aqueous phase decoupling energy ${\Delta G}_{\text{bulk}}^{*}$ = 1.0 kcal mol^−1^, restraint correction ${\Delta G}_{\text{DBC}}$ = 0.9 [sd 0.1 kcal mol^−1^], and volumetric correction ${\Delta G}_{V}^{\circ }$ = 0.4 kcal mol^−1^. For closed-state RyR1, ${\Delta G}_{\text{bind}}^{\circ }$ = –8.4 kcal mol^−1^ [se 0.1 kcal mol^−1^], corresponding to *K*_D_ = 1.4 μM (95% CI: 1.0–2.0 μM), with ${\Delta G}_{\text{DBC}}$ = 1.6 kcal mol^−1^ [sd 0.1 kcal mol^−1^] and the same ${\Delta G}_{\text{bulk}}^{*}$ and ${\Delta G}_{V}^{\circ }$. Convergence was excellent (Fig. S1 in the supplementary material). As *K*_D_ depends logarithmically on ΔG, small changes in ΔG lead to large changes in *K*_D_.

## Discussion

Propofol decreases RyR1 open probability in PLBs and HSM and inhibits [^3^H]ryanodine binding to SR vesicles. Photoaffinity labelling identified several propofol binding sites on RyR1. Overall, these data show that propofol both binds RyR1 in specific sites and inhibits pore opening.

The plasma concentration of propofol at human loss of consciousness was estimated^29^ to be ∼10 μM, within an order of magnitude of our IC_50_ for both propofol inhibition of RyR1 in HSM and predicted binding affinity in the V4828 pocket. Prior studies showed RyR1 inhibition only at high propofol concentrations, which we believe is a result of incomplete solubilisation and lack of free propofol concentration measurements, allowing the possibility of differential binding between sites and unknown allosteric interactions among them.^30^, ^31^, ^32^ All our different approaches agree with respect to concentration dependence. As propofol avidly binds serum proteins,^33^ the plasma concentration required to elicit RyR1 inhibition would be higher than in an otherwise protein-free environment as with our HSM experiments.

In all four subunits, the RyR1 TMD has at least six transmembrane helices of which four encode a (pseudo-)voltage-sensing domain, similar to the inositol-3,4,5-triphosphate (IP3) receptor and voltage-gated ion channels in the Na_v_, K_v_, and Ca_v_ families. Propofol inhibition of channels with TMDs similar to that of the RyR1 is not unique.^34^, ^35^, ^36^ Moreover, binding in the conserved S4–5 linker domain appears to be a canonical feature in ion channels such as NaChBac, Na_v_Ms, and K_v_1.2.^34^^,^^37^ Because propofol binding sites are distributed across this enormous protein, we hypothesise that an allosteric mechanism at least partly underlies its effects. For example, disruption of subunit cooperativity may be important for the function of RyR2.^38^

This study has some limitations. As we did not evaluate a functional model of MH using intact muscle, our prediction that propofol inhibits the clinical presentation of MH is hypothetical and may be trigger-dependent. For example, propofol was unable to reverse heat generation in MH pigs exposed to a high concentration of halothane, a strong trigger.^39^ Further, AziP*m* might adduct sites that the parent ligand propofol does not bind, or *vice versa*, as the diazirine group and halogens render it chemically distinct from the parent ligand, and because the RyR1 sequence was incompletely covered. However, at least in apoferritin, photolabelling, crystallography and fluorescence competition placed AziP*m* and propofol in the same site.^26^ It is reassuring that identical or highly analogous residues were adducted in RyR1 purified from three different sources, and no apparent photochemical selectivity for specific residues was observed.

We compared photoadduction in wild-type and the only readily available mutant protein, pig R615C RyR1. Despite substantial global conformational changes induced by the mutation,^9^ the photoadducted residues were the same with one exception (L2068 in WT *vs* R2072 in R615C). This suggests that the presence of the R615C mutation decreases the energy barrier for RyR1 activation, rather than altering anaesthetic binding.

With the caveat that true *in vivo* RyR1 effect-site concentrations resulting from clinically relevant propofol doses are unknown and therefore not directly comparable to IC_50_ and *K*_D_ values reported here, our results invite the hypothesis that clinical manifestations of MH would be actively inhibited by propofol boluses or infusions, subject to its pharmacokinetics. Skeletal muscle weakness from RyR1 inhibition could conceivably result from propofol administration in both wild-type and mutant RyR1, which might partly explain clinically observed muscle relaxation. However, results from human testing are mixed.^40^^,^^41^

Our findings potentially transfer to other disease states arising from increased RyR1 activation. For example, calcium dysregulation could be an upstream mechanism in neurodegenerative disorders. As all three RyR1 subtypes: RyR1, RyR2, and RyR3, are present in neuronal endoplasmic reticulum, RyR channel dysfunction is correlated to disease progression.^42^, ^43^, ^44^ Thus, in addition to potential salutary effects in MH, this invites the hypothesis that a propofol-based anaesthetic is less likely to aggravate these disorders than known triggering anaesthetics.

## Authors’ contributions

Study design: RGE, FVP, GB, TTJ

Experimental conduct: WB, KW, OHG, YSC

Data analysis: WB, KW, OHG, YSC, TTJ

Simulations: TTJ

Writing the manuscript: TTJ, FVP, PA, RGE

## Acknowledgements

We are grateful to Francisco J. Alvarado (University of Wisconsin-Madison, Madison, WI, USA) for assistance with the [^3^H]ryanodine binding assay.

## Declaration of interest

The authors declare that they have no conflicts of interest.

## Funding

National Institute of General Medical Sciences, US 10.13039/100000002National Institutes of Health (R01GM135633 to RGE, FVP, TTJ; K08GM139031 to TTJ). TTJ was supported by the 10.13039/100005831Foundation for Anesthesia Education and Research (MRTG-BS-Joseph).
