# Supplementary material for: Propofol binds and inhibits skeletal muscle ryanodine receptor 1
Source: Br J Anaesth. 2024 Sep 19;133(5):1093–100. doi: 10.1016/j.bja.2024.06.048 (PMC11488158; doi:10.1016/j.bja.2024.06.048)
Supplement: Multimedia component 1 [file mmc1.docx]

**Supplementary Information**

**Supplementary Figure S1.** Plots indicating various aspects of convergence of free energy MD simulations of propofol in V4828 site in open- and closed-state RyR1. For panels a through e, protein bound state decoupling in left and middle columns, and bulk aqueous phase decoupling in rightmost column.

1. Cumulative sum of ∆G for each window.
2. Per-window ∆G, not summed.
3. Discrepancy in ∆G for each window, between forward (λ increasing) and backward (λ decreasing) directions. A kernel density estimation (KDE) of the probability distribution of these values is shown on the right portion of this subpanel. Smaller values imply better convergence.
4. Discrepancy in ∆G between first and last half of samples for each window. Smaller values imply better convergence.
5. Convergence plot, depicting what fraction of simulation time (x-axis) is necessary to achieve a particular magnitude of discrepancy between forward and backward sampling (y-axis).
6. Cumulative and individual ∆G for the thermodynamic integration (TI) calculation to calculate the energetic cost of the DBC restraint. For open-state RyR1 (left), a 6 Å DBC threshold was used, and for closed-state (middle), a 4 Å DBC threshold was used.
7. Titration curve, showing fraction of protein sites occupied as a function of propofol concentration. Open-state on left, closed-state in middle.

| **Domain** | **Rabbit RyR1 photolabeled with 5 µM AziP*m*** | **Rabbit RyR1 photolabeled with 5 µM AziP*m* in the presence of 200 µM propofol** |
| --- | --- | --- |
| Cyto-plasmic | SLLE**T^2069^**VR (11.90%) |  |
|  | CAPE**M^2440^**HLIQAGK (9.09%) |  |
|  | YL**C^2555^**LAVLPLITK (8.11%) |  |
| Core Solenoid | LRPALGEC**L^3193^**AR (7.89%) | LRPALGEC**L^3193^**AR (8.21%) |
|  | DEFSV**L^3402^**CR (8.09%) | DEFSV**L^3402^**CR (8.36%) |
|  | **M^3638^**TPLYNLPTHR (8.82%) |  |
|  | GETGAMVSST**L^3798^**K (4.67%) |  |
|  | QMVDMLVESSSNVEM**I^4058^**LK (6.06%) |  |
|  | RQFIF**D^4220^**VVNEGGESEK (5.26%) |  |
| Trans-membrane | FLN**Y^4554^**LSR (11.11%) |  |
|  | NFYTLRFLAL**F^4568^**LAFAINFILLFYK (6.67%) |  |
|  | LVLNTPSFPSN**Y^4715^**WDK (8.11%) |  |
|  | **I^4737^**AELLGMDLASLEITAHNER (7.55%) |  |
|  | TI**L^4827^**SSVTHNGK (10.53%) |  |
|  | QLVMTVGLLAVVVY**L^4850^**YTVVAFNFFR (9.09%) |  |

**Supplementary Table S1.** AziP*m* Adducted rabbit RyR1 WT peptides in both conditions (rRyR1 photolabeled with 5 µM AziP*m,* and rRyR1 photolabeled with 5 µM AziP*m* in the presence of 200 µM propofol). Adducted residues are bold, with their residue IDs superscripted. The spectral count ratio of adducted to non-adducted peptide are in parentheses adjacent to each peptide entry.

| **Domain** | **Pig RyR1 photolabeled with 5 µM AziP*m*** | **Pig RyR1**  **photolabeled 5 µM AziP*m* in the presence of 200 µM propofol** |
| --- | --- | --- |
| Cyto-plasmic | VAHALCSH**V^1689^**DQAQLLHALEDAHLPGPLR (4.55%) |  |
|  | LMSL**L^2068^**EK (7.55%) |  |
|  | SLLIVQMGPQEENLMIQS**I^2183^**GNIMNNK (4.92%) |  |
|  | **L^3189^**RPALGECLAR (7.32%) | **L^3189^**RPALGECLAR (7.78%) |
|  | YL**C^2555^** LAVLPLITK (5%) |  |
| Core Solenoid | **M^3634^**TPLYNLPTHR (8.16%) |  |
|  | GETGAMVSST**L^3793^**K (9.45%) |  |
|  | QMVDMLVESSSNVEM**I^4053^**LK (10.23%) |  |
|  | RQF**I^4213^**FDVVNEGGESEK (10.20% |  |
| Trans-membrane | FLN**Y**L^4553^SR (11.63%) |  |
|  | NFYTLRFLALFL**A^4572^**FAINFILLFYK  (9.52%) |  |
|  | LVLNTPSFPSN**Y^4713^**WDK (10.53%) |  |
|  | **I^4735^**AELLGMDLASLEITAHNER (5.88%) |  |
|  | TILSS**V^4828^**THNGK (12.19%) |  |
|  | QLVMTVGLLAVVVY**L^4848^**YTVVAFNFFR (9.52%) |  |
|  | AGGGIGDEIEDPAGDEYE**L^4909^**YR (10.42%) |  |

**Supplementary Table S2.** AziP*m* Adducted pig RyR1 WT peptides in both conditions (pRyR1 photolabeled with 5 µM AziP*m,* and pRyR1 photolabeled with 5 µM AziP*m* in the presence of 200 µM propofol). Adducted residues are bold, with their residue IDs superscripted. The spectral count ratio of adducted to non-adducted peptide are in parentheses adjacent to each peptide entry.

| **Domain** | **Pig RyR1 R615C photolabeled with 5 µM AziP*m*** | **Pig RyR1 R615C**  **photolabeled 5 µM AziP*m* in the presence of 200 µM propofol** |
| --- | --- | --- |
| Cyto-plasmic | VAHALCSH**V^1689^**DQAQLLHALEDAHLPGPLR (5%) |  |
|  | LMSLLEKV**R^2072^** (9.38%) |  |
|  | SLLIVQMGPQEENLMIQS**I^2183^**GNIMNNK (4.26%) |  |
|  | **L^3189^**RPALGECLAR (5.16%) | **L^3189^**RPALGECLAR (6.92%) |
|  | YL**C^2555^** LAVLPLITK (8.3%) |  |
| Core Solenoid | **M^3634^**TPLYNLPTHR (11.52%) |  |
|  | GETGAMVSST**L^3793^**K (8.82%) |  |
|  | QMVDMLVESSSNVEM**I^4053^**LK (8.51%) |  |
|  | RQF**I^4213^**FDVVNEGGESEK (10.52%) |  |
| Trans-membrane | FLNY**L^4553^**SR (10.71%) |  |
|  | NFYTLRFLALFL**A^4572^**FAINFILLFYK (8.69%) |  |
|  | LVLNTPSFPSN**Y^4713^**WDK (9.38%) |  |
|  | **I^4735^**AELLGMDLASLEITAHNER (5.89%) |  |
|  | TLRT**I^4824^**LSSVTHNGK (10.53%)  TILSS**V^4828^**THNGK (10.34%) |  |
|  | QLVMTVGLLAVVVY**L^4848^**YTVVAFNFFR (10.53%) |  |
|  | AGGGIGDEIEDPAGDEYE**L^4909^**YR (6.06%) |  |

**Supplementary Table S3.** AziP*m* Adducted pig RyR1 R615C peptides in both conditions (pRyR1 R615C photolabeled with 5 µM AziP*m,* and pRyR1 R615C photolabeled with 5 µM AziP*m* in the presence of 200 µM propofol). Adducted residues are bold, with their residue IDs superscripted. The spectral count ratio of adducted to non-adducted peptide are in parentheses adjacent to each peptide entry.

**Supplementary Figure S2: Coverage map for rRyR1 mass spectrometry analysis.** Sequence of the purified rabbit ryanodine receptor 1 with high confidence coverage in the mass spectrometry analysis denoted as bold residue codes; this represents 83% coverage.

**MGDGGEGEDEVQFLRTDDEVVLQCSATVLK**EQLKLC**LAAEGFGNRLCFLEPTSNAQNVPP**

**DLAICCFTLEQSLSVRALQEMLANTVEAGVESSQGGGHRTLLYGHAILLRHAHSRMYLSC**

**LTTSR**SMTDK**LAFDVGLQEDATGEACWWTMHPASK**QRSEGEK**VRVGDDLILVSVSSERYL**

**HLSTASGELQVDASFMQTLWNMNPICSCCEEGYVTGGHVLRLFHGHMDECLTISAADSDD**

**QRRLVYYEGGAVCTHARSLWRLEPLRISWSGSHLRWGQPLRIRHVTTGRYLALTEDQGLV**

**VVDACK**AHTK**ATSFCFRVSKEKLDTAPKRDVEGMGPPEIKYGESLCFVQHVASGLWLTYA**

**APDPKALRLGVLKKKAILHQEGHMDDALFLTRCQQEESQAARMIHSTAGLYNQFIKGLDS**

**FSGKPRGSGPPAGPALPIEAVILSLQDLIGYFEPPSEELQHEEKQSK**LRSLRNR**QSLFQE**

**EGMLSLVLNCIDRLNVYTTAAHFAEYAGEEAAESWKEIVNLLYELLASLIR**GNR**ANCALF**

**STNLDWVVSKLDRLEASSGILEVLYCVLIESPEVLNIIQENHIKSIISLLDKHGR**NHK**VL**

**DVLCSLCVCNGVAVRSNQDLITENLLPGRELLLQTNLINYVTSIRPNIFVGRAEGSTQYG**

**KWYFEVMVDEVVPFLTAQATHLRVGWALTEGYSPYPGGGEGWGGNGVGDDLYSYGFDGLH**

**LWTGHVARPVTSPGQHLLAPEDVVSCCLDLSVPSISFRINGCPVQGVFEAFNLDGLFFPV**

**VSFSAGVK**VR**FLLGGRHGEFKFLPPPGYAPCHEAVLPR**ER**LRLEPIKEYR**REGPR**GPHLV**

**GPSRCLSHTDFVPCPVDTVQIVLPPHLER**IR**EKLAENIHELWALTRIEQGWTYGPVRDDN**

**KRLHPCLVNFHSLPEPERNYNLQMSGETLKTLLALGCHVGMADEKAEDNLKK**TKLPK**TYM**

**MSNGYKPAPLDLSHVRLTPAQTTLVDRLAENGHNVWARDRVAQGWSYSAVQDIPARR**NPR

**LVPYRLLDEATKRSNRDSLCQAVRTLLGYGYNIEPPDQEPSQVENQSRWDRVR**IFRAEKS

**YTVQSGRWYFEFEAVTTGEMRVGWARPELRPDVELGADELAYVFNGHRGQRWHLGSEPFG**

**RPWQSGDVVGCMIDLTENTIIFTLNGEVLMSDSGSETAFREIEIGDGFLPVCSLGPGQVG**

**HLNLGQDVSSLRFFAICGLQEGFEPFAINMQRPVTTWFSKSLPQFEPVPPEHPHYEVARM**

**DGTVDTPPCLRLAHRTWGSQNSLVEMLFLRLSLPVQFHQHFRCTAGATPLAPPGLQPPAE**

**DEARAAEPDPDYENLRRSAGGWGEAEGGKEGTAKEGTPGGTPQPGVEAQPVRAENEKDAT**

**TEK**NKK**RGFLFK**AK**KAAMMTQPPATPALPRLPHDVVPADNRDDPEIILNTTTYYYSVRVF**

**AGQEPSCVWVGWVTPDYHQHDMNFDLSKVRAVTVTMGDEQGNVHSSLKCSNCYMVWGGDF**

**VSPGQQGRISHTDLVIGCLVDLATGLMTFTANGKESNTFFQVEPNTKLFPAVFVLPTHQN**

**VIQFELGKQKNIMPLSAAMFLSERKNPAPQCPPRLEVQMLMPVSWSRMPNHFLQVETRR**A

GER**LGWAVQCQDPLTMMALHIPEENRCMDILELSERLDLQRFHSHTLRLYRAVCALGNNR**

**VAHALCSHVDQAQLLHALEDAHLPGPLRAGYYDLLISIHLESACR**SR**RSMLSEYIVPLTP**

**ETRAITLFPPGRK**GGNAR**RHGLPGVGVTTSLRPPHHFSPPCFVAALPAAGVAEAPARLSP**

**AIPLEALRDK**ALR**MLGEAVRDGGQHARDPVGGSVEFQFVPVLKLVSTLLVMGIFGDEDVK**

**QILK**MIEPEVFTEEEEEEEEEEEEEEEEEEDEEEKEEDEEEEEK**EDAEKEEEEAPEGEKE**

**DLEEGLLQMKLPESVKLQMCNLLEYFCDQELQHRVESLAAFAERYVDKLQANQRSRYALL**

**MRAFTMSAAETAR**RTREFR**SPPQEQINMLLHFKDEADEEDCPLPEDIRQDLQDFHQDLLA**

**HCGIQLEGEEEEPEEETSLSSR**LR**SLLETVRLVKKKEEKPEEELPAEEKKPQSLQELVSH**

**MVVRWAQEDYVQSPELVRAMFSLLHRQYDGLGELLRALPRAYTISPSSVEDTMSLLECLG**

**QIRSLLIVQMGPQEENLMIQSIGNIMNNKVFYQHPNLMRALGMHETVMEVMVNVLGGGET**

**K**EIRFPK**MVTSCCRFLCYFCR**ISRQNQR**SMFDHLSYLLENSGIGLGMQGSTPLDVAAASV**

**IDNNELALALQEQDLEKVVSYLAGCGLQSCPMLLAKGYPDIGWNPCGGERYLDFLRFAVF**

**VNGESVEENANVVVRLLIRKPECFGPALRGEGGSGLLAAIEEAIRISEDPARDGPGVR**RD

R**RREHFGEEPPEENRVHLGHAIMSFYAALIDLLGRCAPEMHLIQAGKGEALRIR**AILR**SL**

**VPLDDLVGIISLPLQIPTLGKDGALVQPKMSASFVPDHKASMVLFLDRVYGIENQDFLLH**

**VLDVGFLPDMRAAASLDTATFSTTEMALALNRYLCLAVLPLITKCAPLFAGTEHRAIMVD**

**SMLHTVYR**LSRGRSLTKAQR**DVIEDCLMALCRYIRPSMLQHLLRRLVFDVPILNEFAKMP**

**LKLLTNHYER**CWK**YYCLPTGWANFGVTSEEELHLTRKLFWGIFDSLAHKKYDQELYRMAM**

**PCLCAIAGALPPDYVDASYSSK**AEK**KATVDAEGNFDPRPVETLNVIIPEKLDSFINKFAE**

**YTHEKWAFDKIQNNWSYGENVDEELKTHPMLRPYKTFSEKDKEIYRWPIKESLKAMIAWE**

**WTIEKAREGEEERTEK**KKTR**KISQTAQTYDPREGYNPQPPDLSGVTLSRELQAMAEQLAE**

**NYHNTWGRK**KKQELEAK**GGGTHPLLVPYDTLTAKEK**ARDR**EKAQELLKFLQMNGYAVTRG**

**LKDMELDTSSIEKRFAFGFLQQLLRWMDISQEFIAHLEAVVSSGRVEKSPHEQEIKFFAK**

**ILLPLINQYFTNHCLYFLSTPAKVLGSGGHASNKEKEMITSLFCKLAALVRHRVSLFGTD**

**APAVVNCLHILAR**SLDARTVMK**SGPEIVKAGLRSFFESASEDIEKMVENLR**LGKVSQART

QVK**GVGQNLTYTTVALLPVLTTLFQHIAQHQFGDDVILDDVQVSCYRTLCSIYSLGTTKN**

**TYVEKLRPALGECLARLAAAMPVAFLEPQLNEYNACSVYTTKSPR**ER**AILGLPNSVEEMC**

**PDIPVLDRLMADIGGLAESGARYTEMPHVIEITLPMLCSYLPR**WWER**GPEAPPPALPAGA**

**PPPCTAVTSDHLNSLLGNILRIIVNNLGIDEATWMKRLAVFAQPIVSRARPELLHSHFIP**

**TIGRLRKRAGKVVAEEEQLRLEAKAEAEEGELLVRDEFSVLCRDLYALYPLLIRYVDNNR**

**AHWLTEPNANAEELFRMVGEIFIYWSKSHNFKREEQNFVVQNEINNMSFLTADSKSK**MAK

**AGDAQSGGSDQERTKKKRRGDRYSVQTSLIVATLKKMLPIGLNMCAPTDQDLIMLAKTRY**

**ALKDTDEEVREFLQNNLHLQGKVEGSPSLRWQMALYRGLPGREEDADDPEKIVRRVQEVS**

**AVLYHLEQTEHPYKSKKAVWHKLLSKQRRRAVVACFRMTPLYNLPTHRACNMFLESYKAA**

**WILTEDHSFEDRMIDDLSKAGEQEEEEEEVEEKKPDPLHQLVLHFSRTALTEKSKLDEDY**

**LYMAYADIMAKSCHLEEGGENGEAEEEEVEVSFEEKEMEK**QR**LLYQQSR**LHTR**GAAEMVL**

**QMISACKGETGAMVSSTLKLGISILNGGNAEVQQKMLDYLKDKKEVGFFQSIQALMQTCS**

**VLDLNAFERQNKAEGLGMVNEDGTVINRQNGEKVMADDEFTQDLFRFLQLLCEGHNNDFQ**

**NYLRTQTGNTTTINIIICTVDYLLRLQESISDFYWYYSGKDVIEEQGKRNFSKAMSVAKQ**

**VFNSLTEYIQGPCTGNQQSLAHSRLWDAVVGFLHVFAHMMMKLAQDSSQIELLKELLDLQ**

**KDMVVMLLSLLEGNVVNGMIARQMVDMLVESSSNVEMILKFFDMFLKLKDIVGSEAFQDY**

**VTDPR**GLISKKDFQKAMDSQKQ**FTGPEIQFLLSCSEADENEMINFEEFANRFQEPARDIG**

**FNVAVLLTNLSEHVPHDPRLRNFLELAESILEYFRPYLGRIEIMGASR**RIER**IYFEISET**

**NRAQWEMPQVKESKRQFIFDVVNEGGEAEK**MELFVSFCEDTIFEMQIAAQISEPEGEPEA

DEDEGMGEAAAEGAEEGAAGAEGAAGTVAAGATAR**LAAAAARALRGLSYR**SLRRRVRRLR

**RLTAREAATALAALLWAVVARAGAAGAGAAAGALRLLWGSLFGGGLVEGAKK**VTVTELLA

GMPDPTSDEVHGEQPAGPGGDADGAGEGEGEGDAAEGDGDEEVAGHEAGPGGAEGVVAVA

DGGPFRPEGAGGLGDMGDTTPAEPPTPEGSPILKR**KLGVDGEEEELVPEPEPEPEPEPEK**

**ADEENGEKEEVPEAPPEPPKKAPPSPPAKKEEAGGAGMEFWGELEVQRVKFLNYLSRNFY**

**TLRFLALFLAFAINFILLFYK**VSDSPPGEDDMEGSAAGDLAGAGSGGGSGWGSGAGEEAE

GDEDENMVYYFLEESTGYMEPALWCLSLLHTLVAFLCIIGYNCLK**VPLVIFKR**EKELAR**K**

**LEFDGLYITEQPGDDDVKGQWDRLVLNTPSFPSNYWDKFVKRKVLDKHGDIFGRERIAEL**

**LGMDLASLEITAHNERKPDPPPGLLTWLMSIDVK**YQIWKFGVIFTDNSFLYLGWYMVMSL

LGHYNNFFFAAHLLDIAMGVK**TLRTILSSVTHNGKQLVMTVGLLAVVVYLYTVVAFNFFR**

**KFYNKSEDEDEPDMKCDDMMTCYLFHMYVGVRAGGGIGDEIEDPAGDEYELYR**VVFDITF

FFFVIVILLAIIQGLIIDAFGELR**DQQEQVKEDMETKCFICGIGSDYFDTTPHGFETHTL**

**EEHNLANYMFFL**M**YLINKDETEHTGQESYVWK**MYQER**CWDFFPAGDCFRKQYEDQLS**

**Supplemental Figure S3: Coverage map for Pig RyR1 mass spectrometry analysis.** Sequence of the purified rabbit RyR 1 with high confidence coverage in mass spectrometry analysis are bolded; this represents 87.5% coverage.

**MGDGGEGEDEVQFLRTDDEVVLQCNATVLK**EQLK**LCLAAEGFGNR**LCFLEPTSNAQNVPP

DLAICCFVLEQSLSVR**ALQEMLANTVEAGVESSQGGGHRTLLYGHAILLRHAHSGMYLSC**

**LTTSR**SMTDK**LAFDVGLQEDATGEACWWTTHPASK**QRSEGEK**VRVGDDLILVSVSSER**YL

HLSTASGELQVDASFMQTLWNMNPICSGCEEGYVTGGHVLR**LFHGHMDECLTISPADSDD**

**QRRLVYYEGGSVCTHARSLWRLEPLRISWSGSHLRWGQPLRIRHVTTGRYLALIEDQGLV**

**VVDASKAHTKATSFCFRISKEKLDTAPKRDVEGMGPPEIKYGESLCFVQHVASGLWLTYA**

**APDPKALRLGVLKKKAILHQEGHMDDALSLTRCQQEESQAARMIYSTAGLYNHFIKGLDS**

**FSGKPRGSGAPAGTALPLEGVILSLQDLIGYFEPPSEELQHEEK**QSK**L**RSLRNR**QSLFQE**

**EGMLSLVLNCIDRLNVYTTAAHFAEFAGEEAAESWKEIVNLLYEILASLIRGNRANCALF**

**SNNLDWLVSK**LDR**LEASSGILEVLYCVLIESPEVLNIIQENHIKSIISLLDKHGR**NHKVL

DVLCSLCVCNGVAVR**SNQDLITENLLPGRELLLQTNLINYVTSIRPNIFVGRAEGTTQYS**

**KWYFEVMVDEVVPFLTAQATHLRVGWALTEGYSPYPGGGEGWGGNGVGDDLYSYGFDGLH**

**LWTGHVPRLVTSPGQHLLAPEDVVSCCLDLSVPSISFRINGCPVQGVFEAFNLNGLFFPV**

**VSFSAGVKVRFLLGGRHGEFKFLPPPGYAPCHEAVLPR**ER**LRLEPIKEYRR**EGPR**GPHLV**

**GPSRCLSHTDFVPCPVDTVQIVLPPHLERIREKLAENIHELWALTRIEQGWTYGPVRDDN**

**KRLHPCLVDFHSLPEPERNYNLQMSGETLKTLLALGCHVGMADEKAEDNLRK**TKLPK**TYM**

**MSNGYKPAPLDLSHVRLTPAQTTLVDRLAENGHNVWARDRVAQGWSYSAVQDIPARRNPR**

**LVPYRLLDEATKRSNRDSLCQAVRTLLGYGYNIEPPDQEPSQVESQSRWDR**VR**IFRAEKS**

**YAVQSGRWYFEFEAVTTGEMRVGWARPELRPDVELGADELAYVFNGHRGQRWHLGSELFG**

RPWQSGDVVGCMIDLTENTIIFTLNGEVLMSDSGSETAFR**DIEVGDGFLPVCSLGPGQVG**

**HLNLGQDVSSLRFFAICGLQEGFEPFAINMQRPVTTWFSKSLPQFEAVPLEHPHYEVSRV**

**DGTVDTPPCLRLTHRTWGSQNSLVEMLFLRLSLPVQFHQHFRCTAGATPLAPPGLQPPAE**

**DEARAAEPDPDYENLRRSAGRWGEAEGGKEGTAKEGAPGGTAQAGVEAQPPRAENEKDAT**

**TEKNK**KR**GFLFKAKKAAMMTQPPATPTLPRLPHEVVPADDRDDPDIILNTTTYYYSVRVF**

**AGQEPSCVWVGWVTPDYHQHDMNFDLTK**VR**AVTVTMGDEQGNIHSSLKCSNCYMVWGGDF**

**VSPGQQGRISHTDLVIGCLVDLATGLMTFTANGKESNTFFQVEPNTKLFPAVFVLPTHQN**

**VIQFELGKQKNIMPLSAAMFLSERKNPAPQCPPRLEMQMLMPVSWSRMPNHFLRVETR**RA

GER**LGWAVQCQEPLTMMALHIPEENRCMDILELSERLDLQQFHSHTLRLYRAVCALGNNR**

**VAHALCSHVDQAQLLHALEDAHLPGPLRAGYYDLLISIHLESACR**SR**RSMLSEYIVPLTP**

**ETRAITLFPPGKR**TENGPR**RHGLPGVGVTTSLRPPHHFSAPCFVAALPAVGAAEAPARLS**

**PSIPLEALRDKALRMLGEAVRDGGQHARDPVGGSVEFQFVPVLKLVSTLLVMGIFGDEDV**

**KQILKMIEPEVFTEEEEEEEEEEEEEEEDEEEKEEDEEEEAREKEDEEKEEEETAEGEKE**

**EYLEEGLLQMK**LPESVK**LQMCNLLEYFCDQELQHRVESLAAFAERYVDKLQANQRDRYGI**

**LMKAFTMTAAETARR**TREFR**SPPQEQINMLLHFKDGEDEEDCPLPDEIRQDLLEFHQDLL**

**THCGIQLEGEEEEPEEEATLGSRLMSLLEKVR**LVKK**KEEKSEEEPPAEESK**AQSLQELVS

HTVVR**WAQEDFVQSPELVRAMFSLLHRQYDGLGELLRALPRAYTISPSSVEDTMSLLECL**

**GQIRSLLIVQMGPQEENLMIQSIGNIMNNKVFYQHPNLMRALGMHETVMEVMVNVLGGGE**

**SKEIRFPK**M**VTSCCRFLCYFCR**ISRQNQR**SMFDHLSYLLENSGIGLGMQGSTPLDVAAAS**

**VIDNNELALALQEQDLEKVVSYLAGCGLQSCPMLLAKGYPDIGWNPCGGERYLDFLRFAV**

**FVNGESVEENANVVVRLLIRKPECFGPALRGEGGSGLLATIEEAIRISEDPARDGPGVR**R

DR**RREHFGEEPPEENRVHLGHAIMSFYAALIDLLGRCAPEMHLIQAGKGEALRIRAILRS**

**LVPLDDLVGIISLPLQIPTLGKDGALVQPKMSASFVPDHKASMVLFLDRVYGIENQDFLL**

**HVLDVGFLPDMRAAASLDTATFSTTEMALALNRYLCLAVLPLITKCAPLFAGTEHRAIMV**

**DSMLHTVYRLSR**GRSLTKAQR**DVIEECLMALCRYIRPSMLQHLLRRLVFDVPILNEFAKM**

**PLKLLTNHYER**CWK**YYCLPTGWANFGVTSEEELHLTRKLFWGIFDSLAHKKYDPELYRMA**

**MPCLCAIAGALPPDYVDASYSSK**AEK**KATVDAEGNFDPRPVETLNVIIPEKLDSFINKFA**

**EYTHEKWAFDKIQNNWSYGENIDEELKTHPMLRPYKTFSEKDKEIYRWPIKESLKAMIAW**

**EWTIEKAREGEEEKTEK**KK**TRKISQSAQTYDAREGYNPQPPDLSGVTLSRELQAMAEQLA**

**ENYHNTWGRKKKQELEAKGGGTHPLLVPYDTLTAKEK**ARDR**EKAQELLKFLQMNGYAVTR**

**GLKDMELDTSSIEKRFAFGFLQQLLRWMDISQEFIAHLEAVVSSGRVEKSPHEQEIKFFA**

**KILLPLINQYFTNHCLYFLSTPAKVLGSGGHASNKEKEMITSLFCKLAALVR**HR**VSLFGT**

**DAPAVVNCLHILARSLDARTVMKSGPEIVKAGLRSFFESASEDIEKMVENLRLGKVSQAR**

**TQVKGVGQNLTYTTVALLPVLTTLFQHIAQHQFGDDVILDDVQVSCYRTLCSIYSLGTTR**

**NPYVEKLRPALGECLARLAAAMPVAFLEPQLNEYNACSVYTTK**SPRER**AILGLPNSVEEM**

**CPDIPVLERLMADIGGLAESGARYTEMPHVIEITLPMLCSYLPR**WWER**GPEAPPPALPAG**

**APPPCTAVTSDHLNSLLGNILRIIVNNLGIDEASWMKRLAVFAQPIVSRARPELLHSHFI**

**PTIGRLRKRAGKVVAEEEQLRLEAKAEAEEGELLVRDEFSVLCRDLYALYPLLIRYVDNN**

**RAHWLTEPNPSAEELFRMVGEIFIYWSKSHNFKREEQNFVVQNEINNMSFLTADNKSK**MA

K**SGGSDQER**TKKKRLGDR**YSVQTSLIVATLKKMLPIGLNMCAPTDQELITLAKTRYALKD**

**TDEEVREFLQNNLHLQGKVEGSPSLRWQMALYRGLPGREEDADDPEKIVRRVQEVSAVLY**

**HLEQMEHPYKSKKAVWHKLLSK**QRR**RAVVACFRMTPLYNLPTHRACNMFLESYKAAWILT**

**EDHSFEDRMIDDLSKAGEQEEEEEEVEEKKPDPLHQLVLHFSRTALTEKSKLDEDYLYMA**

**YADIMAKSCHLEEGGENGEAQEEVEVSFEEKEMEKQRLLYQQARLHNRGAAEMVLQMISA**

**CKGETGAMVSSTLKLGISILNGGNADVQQKMLDYLKDKKEVGFFQSIQALMQTCSVLDLN**

**AFERQNKAEGLGMVNEDGTVINRQNGEKVMADDEFTQDLFRFLQLLCEGHNNDFQNYLRT**

**QTGNTTTINIIICTVDYLLRLQESISDFYWYYSGKDVIEEQGKRNFSKAMSVAKQVFNSL**

**TEYIQGPCTGNQQSLAHSRLWDAVVGFLHVFAHMMMKLAQDSSQIELLKELLDLQKDMVV**

**MLLSLLEGNVVNGMIARQMVDMLVESSSNVEMILKFFDMFLKLKDIVGSEAFQDYVTDPR**

**GLISKKDFQKAMDSQKQFTGPEIQFLLSCSEADENEMIDCEEFANRFQEPARDIGFNVAV**

**LLTNLSEHVPHDPRLRNFLELAESILEYFRPYLGRIEIMGASRR**IER**IYFEISETNRAQW**

**EMPQVKESKRQFIFDVVNEGGESEK**MELFVSFCEDTIFEMQIAAQISEPEGEPEEDEDEG

AGLAEAGAEGAEEGAVGPEGAAGTAAAGLTAR**LAAATSRALRGLSYR**SLRRRVRRLRRLT

AR**EAATALAALLWAALAHAGAAGAGAAAGALRLLWGSLFGGGLVEGAKK**VTVTELLAGMP

DPTGDEVHGEQPAGPGGEADGEGAGEGAGEAWEGAGDEEVAVQEAGPGGADGAVAVAEGG

PFRPEGAGGLGDMGDTTPAEPPTPEGSPIIKR**KLGVDGEEEELPPEPEPEPEPEPEKADA**

**ENGEKEEVPKPPPEPPKKTAPPPPPPKKEEGGSGGLEFWGELEVQRVKFLNYLSRNFYTL**

**RFLALFLAFAINFILLFYK**VSDSPPGEDDMEGSAAGDLSGAGSGGGSGWGSGAGEEVEGD

EDENMVYYFLEESTGYMEPALRCLSLLHTLVAFLCIIGYNCLK**VPLVIFKR**EKELAR**KLE**

**FDGLYITEQPEDDDVKGQWDRLVLNTPSFPSNYWDKFVKRKVLDKHGDIYGRERIAELLG**

**MDLATLEITAHNERKPEPPPGLLTWLMSIDVKYQIWKFGVIFTDNSFLYLGWYMVMSLLG**

**HYNNFFFAAHLLDIAMGVK**TLR**TILSSVTHNGKQLVMTVGLLAVVVYLYTVVAFNFFRKF**

**YNKSEDEDEPDMK**CDDMMTCYLFHMYVGVR**AGGGIGDEIEDPAGDEYELYR**VVFDITFFF

FVIVILLAIIQGLIIDAFGELR**DQQEQVREDMETK**CFICGIGSDYFDTTPHRFETHTLEE

HNLANYMFFLMYLINK**DETEHTGQESYVWK**MYQER**CWDFFPAGDCFRKQYEDQLS**

**Supplemental Figure S4: Coverage map for Pig RyR1 R615C mass spectrometry analysis** (R615C mutation in red and underlined). Sequence of the purified rabbit ryanodine receptor 1 with high confidence coverage in the mass spectrometry analysis denoted as bold residue codes (representing 80.2% coverage).

**MGDGGEGEDEVQFLRTDDEVVLQCNATVLK**EQLK**LCLAAEGFGNR**LCFLEPTSNAQNVPP

DLAICCFVLEQSLSVRALQEMLANTVEAGVESSQGGGHRTLLYGHAILLR**HAHSGMYLSC**

**LTTSRSMTDKLAFDVGLQEDATGEACWWTTHPASK**QRSEGEK**VRVGDDLILVSVSSERYL**

**HLSTASGELQVDASFMQTLWNMNPICSGCEEGYVTGGHVLRLFHGHMDECLTISPADSDD**

**QRRLVYYEGGSVCTHARSLWRLEPLRISWSGSHLRWGQPLRIRHVTTGRYLALIEDQGLV**

**VVDASKAHTKATSFCFRISKEKLDTAPKRDVEGMGPPEIKYGESLCFVQHVASGLWLTYA**

**APDPKALRLGVLKKKAILHQEGHMDDALSLTR**CQQEESQAAR**MIYSTAGLYNHFIKGLDS**

**FSGKPRGSGAPAGTALPLEGVILSLQDLIGYFEPPSEELQHEEKQSKL**RSLRNR**QSLFQE**

**EGMLSLVLNCIDRLNVYTTAAHFAEFAGEEAAESWKEIVNLLYEILASLIRGNRANCALF**

**SNNLDWLVSKLDRLEASSGILEVLYCVLIESPEVLNIIQENHIKSIISLLDKHGR**NHKVL

DVLCSLCVCNGVAV**C**SNQDLITENLLPGR**ELLLQTNLINYVTSIRPNIFVGRAEGTTQYS**

**KWYFEVMVDEVVPFLTAQATHLRVGWALTEGYSPYPGGGEGWGGNGVGDDLYSYGFDGLH**

**LWTGHVPRLVTSPGQHLLAPEDVVSCCLDLSVPSISFRINGCPVQGVFEAFNLNGLFFPV**

**VSFSAGVKVRFLLGGR**HGEFK**FLPPPGYAPCHEAVLPR**ER**LRLEPIKEYRR**EGPR**GPHLV**

**GPSRCLSHTDFVPCPVDTVQIVLPPHLERIREKLAENIHELWALTRIEQGWTYGPVRDDN**

**KRLHPCLVDFHSLPEPERNYNLQMSGETLKTLLALGCHVGMADEKAEDNLRK**TK**LPKTYM**

**MSNGYKPAPLDLSHVRLTPAQTTLVDRLAENGHNVWARDRVAQGWSYSAVQDIPARRNPR**

**LVPYRLLDEATKRSNRDSLCQAVRTLLGYGYNIEPPDQEPSQVESQSRWDR**VR**IFRAEKS**

**YAVQSGRWYFEFEAVTTGEMRVGWARPELRPDVELGADELAYVFNGHRGQRWHLGSELFG**

**RPWQSGDVVGCMIDLTENTIIFTLNGEVLMSDSGSETAFRDIEVGDGFLPVCSLGPGQVG**

**HLNLGQDVSSLRFFAICGLQEGFEPFAINMQRPVTTWFSKSLPQFEAVPLEHPHYEVSRV**

**DGTVDTPPCLRLTHRTWGSQNSLVEMLFLRLSLPVQFHQHFRCTAGATPLAPPGLQPPAE**

**DEARAAEPDPDYENLRRSAGRWGEAEGGKEGTAKEGAPGGTAQAGVEAQPPRAENEKDAT**

**TEK**NKK**RGFLFKAKKAAMMTQPPATPTLPRLPHEVVPADDRDDPDIILNTTTYYYSVRVF**

**AGQEPSCVWVGWVTPDYHQHDMNFDLTK**VR**AVTVTMGDEQGNIHSSLKCSNCYMVWGGDF**

**VSPGQQGRISHTDLVIGCLVDLATGLMTFTANGKESNTFFQVEPNTKLFPAVFVLPTHQN**

**VIQFELGKQKNIMPLSAAMFLSERKNPAPQCPPRLEMQMLMPVSWSRMPNHFLR**VETRRA

GER**LGWAVQCQEPLTMMALHIPEENRCMDILELSERLDLQQFHSHTLRLYRAVCALGNNR**

**VAHALCSHVDQAQLLHALEDAHLPGPLRAGYYDLLISIHLESACR**SR**RSMLSEYIVPLTP**

**ETRAITLFPPGKR**TENGPR**RHGLPGVGVTTSLRPPHHFSAPCFVAALPAVGAAEAPARLS**

**PSIPLEALRDKALRMLGEAVRDGGQHARDPVGGSVEFQFVPVLKLVSTLLVMGIFGDEDV**

**KQILKMIEPEVFTEEEEEEEEEEEEEEEDEEEKEEDEEEEAREKEDEEKEEEETAEGEKE**

**EYLEEGLLQMKLPESVKLQMCNLLEYFCDQELQHRVESLAAFAERYVDKLQANQRDRYGI**

**LMKAFTMTAAETARR**TREFR**SPPQEQINMLLHFKDGEDEEDCPLPDEIRQDLLEFHQDLL**

**THCGIQLEGEEEEPEEEATLGSRLMSLLEKVR**LVKKKEEK**SEEEPPAEESK**AQSLQELVS

HTVVR**WAQEDFVQSPELVRAMFSLLHRQYDGLGELLRALPRAYTISPSSVEDTMSLLECL**

**GQIRSLLIVQMGPQEENLMIQSIGNIMNNKVFYQHPNLMRALGMHETVMEVMVNVLGGGE**

**SKEIRFPK**MVTSCCR**FLCYFCR**ISRQNQR**SMFDHLSYLLENSGIGLGMQGSTPLDVAAAS**

**VIDNNELALALQEQDLEKVVSYLAGCGLQSCPMLLAKGYPDIGWNPCGGERYLDFLRFAV**

**FVNGESVEENANVVVRLLIRKPECFGPALRGEGGSGLLATIEEAIRISEDPARDGPGVR**R

DR**RREHFGEEPPEENRVHLGHAIMSFYAALIDLLGRCAPEMHLIQAGKGEALRIRAILRS**

**LVPLDDLVGIISLPLQIPTLGKDGALVQPKMSASFVPDHKASMVLFLDRVYGIENQDFLL**

**HVLDVGFLPDMRAAASLDTATFSTTEMALALNRYLCLAVLPLITKCAPLFAGTEHRAIMV**

**DSMLHTVYRLSR**GRSLTKAQR**DVIEECLMALCRYIRPSMLQHLLRRLVFDVPILNEFAKM**

**PLKLLTNHYER**CWK**YYCLPTGWANFGVTSEEELHLTRKLFWGIFDSLAHKKYDPELYRMA**

**MPCLCAIAGALPPDYVDASYSSK**AEK**KATVDAEGNFDPRPVETLNVIIPEKLDSFINKFA**

**EYTHEKWAFDKIQNNWSYGENIDEELKTHPMLRPYKTFSEKDKEIYRWPIKESLKAMIAW**

**EWTIEKAR**EGEEEKTEKKK**TRKISQSAQTYDAREGYNPQPPDLSGVTLSRELQAMAEQLA**

**ENYHNTWGRKKKQELEAKGGGTHPLLVPYDTLTAKEK**AR**DREKAQELLKFLQMNGYAVTR**

**GLKDMELDTSSIEKRFAFGFLQQLLRWMDISQEFIAHLEAVVSSGRVEKSPHEQEIKFFA**

**KILLPLINQYFTNHCLYFLSTPAKVLGSGGHASNKEKEMITSLFCKLAALVR**HR**VSLFGT**

**DAPAVVNCLHILARSLDARTVMKSGPEIVKAGLRSFFESASEDIEKMVENLRLGKVSQAR**

**TQVKGVGQNLTYTTVALLPVLTTLFQHIAQHQFGDDVILDDVQVSCYRTLCSIYSLGTTR**

**NPYVEKLRPALGECLARLAAAMPVAFLEPQLNEYNACSVYTTKSPRERAILGLPNSVEEM**

**CPDIPVLERLMADIGGLAESGARYTEMPHVIEITLPMLCSYLPR**WWER**GPEAPPPALPAG**

**APPPCTAVTSDHLNSLLGNILRIIVNNLGIDEASWMKRLAVFAQPIVSRARPELLHSHFI**

**PTIGRLRKRAGKVVAEEEQLRLEAKAEAEEGELLVRDEFSVLCRDLYALYPLLIRYVDNN**

**RAHWLTEPNPSAEELFRMVGEIFIYWSKSHNFKREEQNFVVQNEINNMSFLTADNKSK**MA

**KSGGSDQERTKKKRLGDRYSVQTSLIVATLKKMLPIGLNMCAPTDQELITLAKTRYALKD**

**TDEEVREFLQNNLHLQGKVEGSPSLRWQMALYRGLPGREEDADDPEKIVRRVQEVSAVLY**

**HLEQMEHPYKSKKAVWHKLLSKQRRRAVVACFRMTPLYNLPTHRACNMFLESYKAAWILT**

**EDHSFEDRMIDDLSKAGEQEEEEEEVEEKKPDPLHQLVLHFSRTALTEKSKLDEDYLYMA**

**YADIMAKSCHLEEGGENGEAQEEVEVSFEEKEMEKQRLLYQQAR**LHNR**GAAEMVLQMISA**

**CKGETGAMVSSTLKLGISILNGGNADVQQKMLDYLKDKKEVGFFQSIQALMQTCSVLDLN**

**AFERQNKAEGLGMVNEDGTVINRQNGEKVMADDEFTQDLFRFLQLLCEGHNNDFQNYLRT**

**QTGNTTTINIIICTVDYLLRLQESISDFYWYYSGKDVIEEQGKRNFSKAMSVAKQVFNSL**

**TEYIQGPCTGNQQSLAHSRLWDAVVGFLHVFAHMMMKLAQDSSQIELLKELLDLQKDMVV**

**MLLSLLEGNVVNGMIARQMVDMLVESSSNVEMILKFFDMFLKLKDIVGSEAFQDYVTDPR**

**GLISKKDFQKAMDSQKQFTGPEIQFLLSCSEADENEMIDCEEFANRFQEPARDIGFNVAV**

**LLTNLSEHVPHDPRLRNFLELAESILEYFRPYLGRIEIMGASRRIERIYFEISETNRAQW**

**EMPQVKESKRQFIFDVVNEGGESEK**MELFVSFCEDTIFEMQIAAQISEPEGEPEEDEDEG

AGLAEAGAEGAEEGAVGPEGAAGTAAAGLTAR**LAAATSRALRGLSYR**SLRRRVRRLRR**LT**

**AREAATALAALLWAALAHAGAAGAGAAAGALRLLWGSLFGGGLVEGAKK**VTVTELLAGMP

DPTGDEVHGEQPAGPGGEADGEGAGEGAGEAWEGAGDEEVAVQEAGPGGADGAVAVAEGG

PFRPEGAGGLGDMGDTTPAEPPTPEGSPIIKR**KLGVDGEEEELPPEPEPEPEPEPEKADA**

**ENGEKEEVPKPPPEPPKKTAPPPPPPKKEEGGSGGLEFWGELEVQRVKFLNYLSRNFYTL**

**RFLALFLAFAINFILLFYK**VSDSPPGEDDMEGSAAGDLSGAGSGGGSGWGSGAGEEVEGD

EDENMVYYFLEESTGYMEPALRCLSLLHTLVAFLCIIGYNCLK**VPLVIFKREKELARKLE**

**FDGLYITEQPEDDDVKGQWDRLVLNTPSFPSNYWDKFVKRKVLDKHGDIYGRERIAELLG**

**MDLATLEITAHNERKPEPPPGLLTWLMSIDVKYQIWKFGVIFTDNSFLYLGWYMVMSLLG**

**HYNNFFFAAHLLDIAMGVKTLRTILSSVTHNGKQLVMTVGLLAVVVYLYTVVAFNFFRKF**

**YNKSEDEDEPDMKCDDMMTCYLFHMYVGVRAGGGIGDEIEDPAGDEYELYR**VVFDITFFF

FVIVILLAIIQGLIIDAFGELR**DQQEQVREDMETK**CFICGIGSDYFDTTPHRFETHTLEE

HNLANYMFFLMYLINK**DETEHTGQESYVWK**MYQER**CWDFFPAGDCFRKQYEDQLS**

**Supplementary Figure S5. Mass spectra of AziPm photolabeled rabbit RyR1 peptides.** Residues detected with an AziPm photomodification are bolded. Colored intensities denote the identified peptide b and y ion fragments for the peptide sequence assignment. Identified a+ (light blue), b^+^ (red), b^2+^ (red), y^+^ (blue), and y^2+^ (blue) ions are labeled accordingly. Residues detected with an AziPm photomodification are labeled as “az”.

Figure S5a. SLLE**T^2069^**VR-AziP*m*


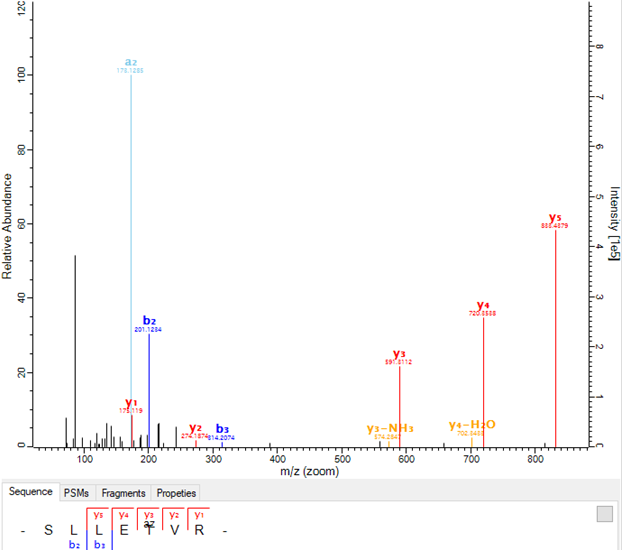


Figure S5b, CAPE**M^2440^**HLIQAGK-AziP*m*


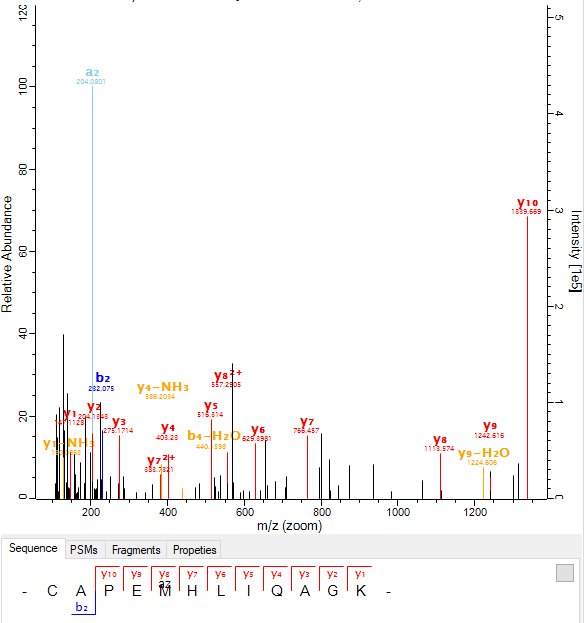


Figure S5c, YL**C^2555^**LAVLPLITK-AziP*m*.


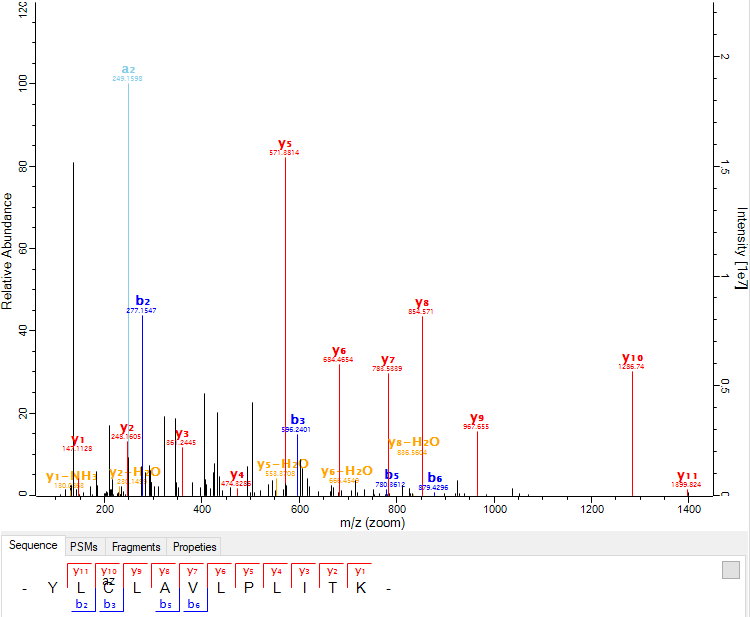


Figure S5d. **M^3638^**TPLYNLPTHR-AziP*m*


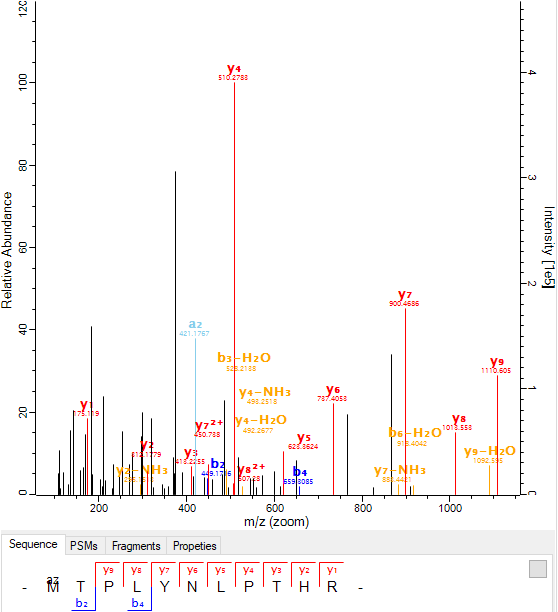


Figure S5e, GETGAMVSST**L^3798^**K-AziP*m*


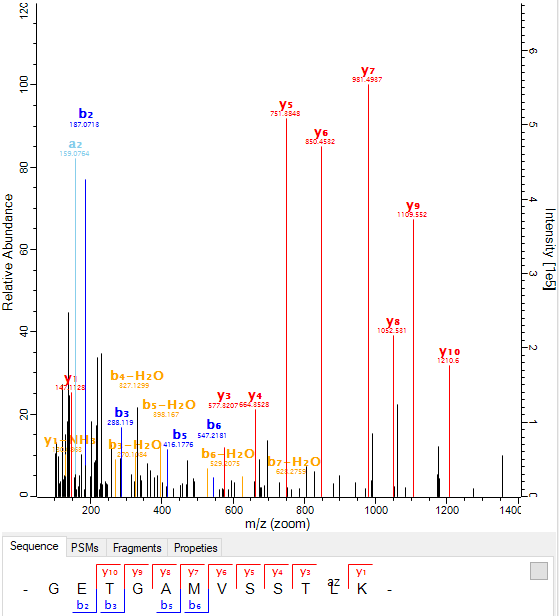


Figure S5f. QMVDMLVESSSNVEM**I^4058^** LK-AziP*m*


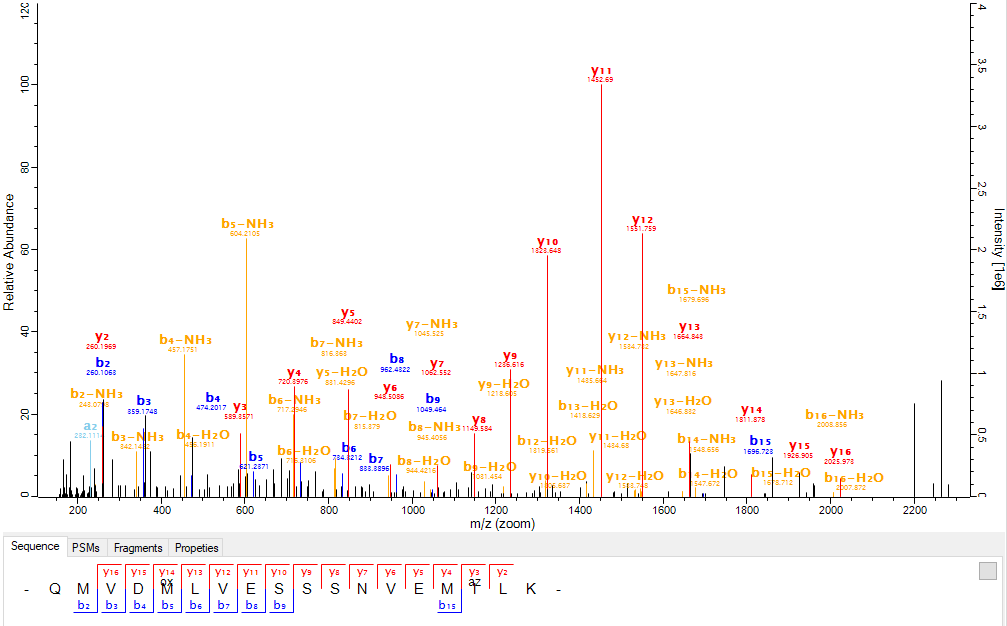


Figure S5g. RQFIF**D^4220^**VVNEGGESEK-AziP*m*


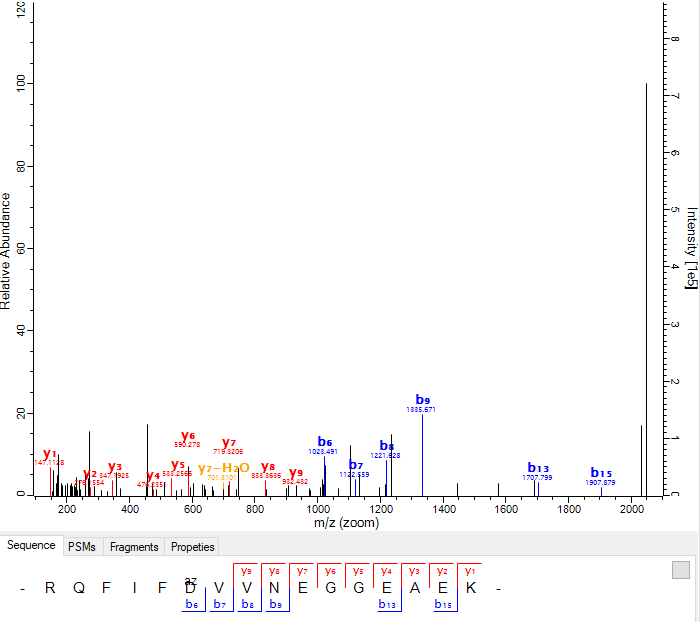


Figure S5h. FLN**Y^4554^**LSR-AziP*m*


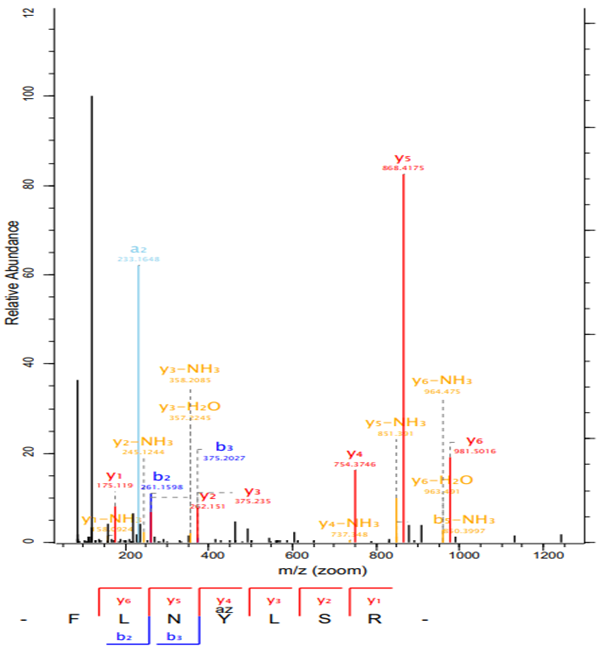


Figure S5i. NFYTLRFLAL**F^4568^** LAFAINFILLFYK-AziP*m*


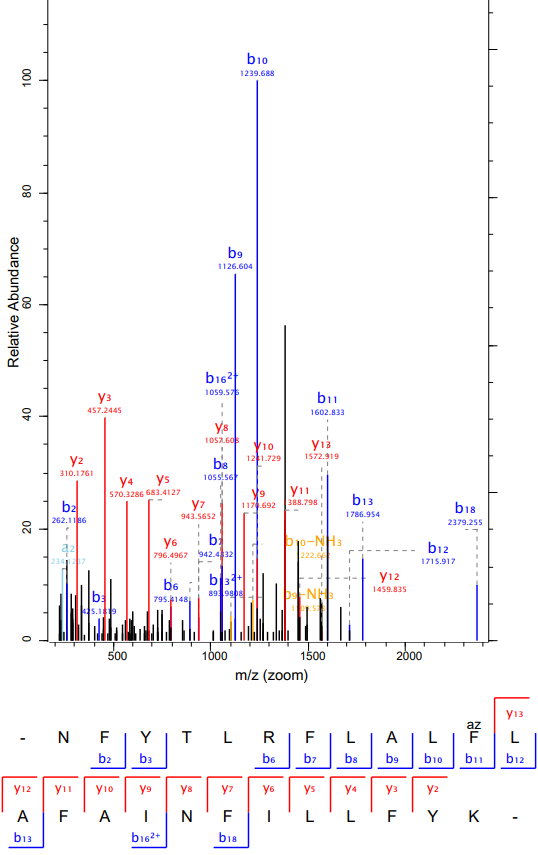


Figure S5j. LVLNTPSFPSN**Y^4715^**WDK-AziP*m*


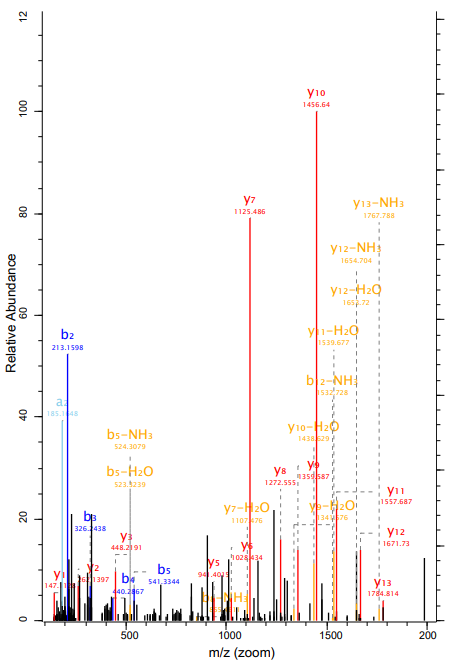


Figure S5k**. I^4737^**AELLGMDLASLEITAHNER-AziP*m*


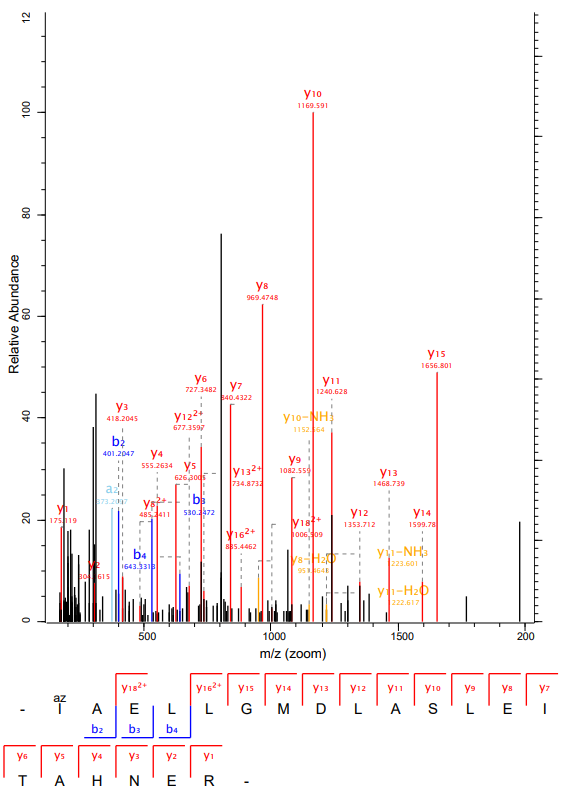


Figure S5l. TI**L^4827^**SSVTHNGK-AziP*m*


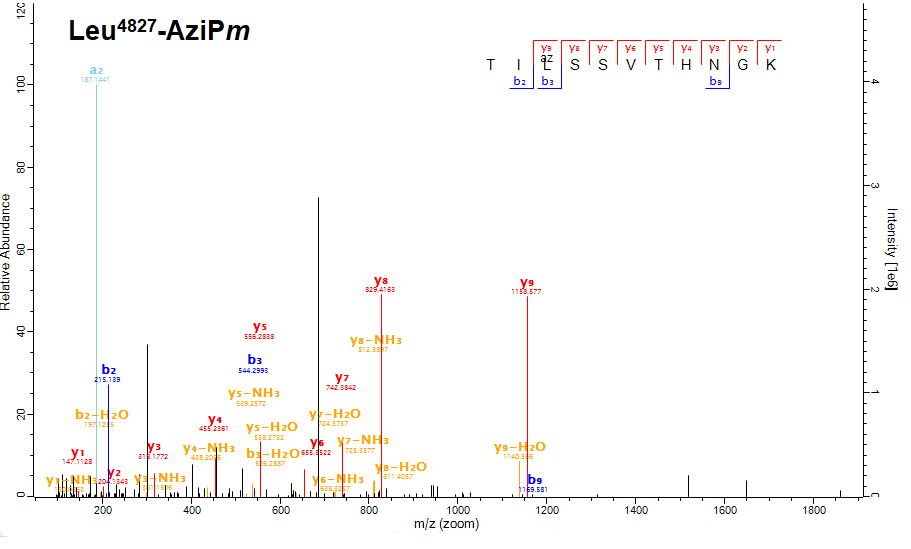


Figure S5m. QLVMTVGLLAVVVY**L^4850^**YTVVAFNFFR


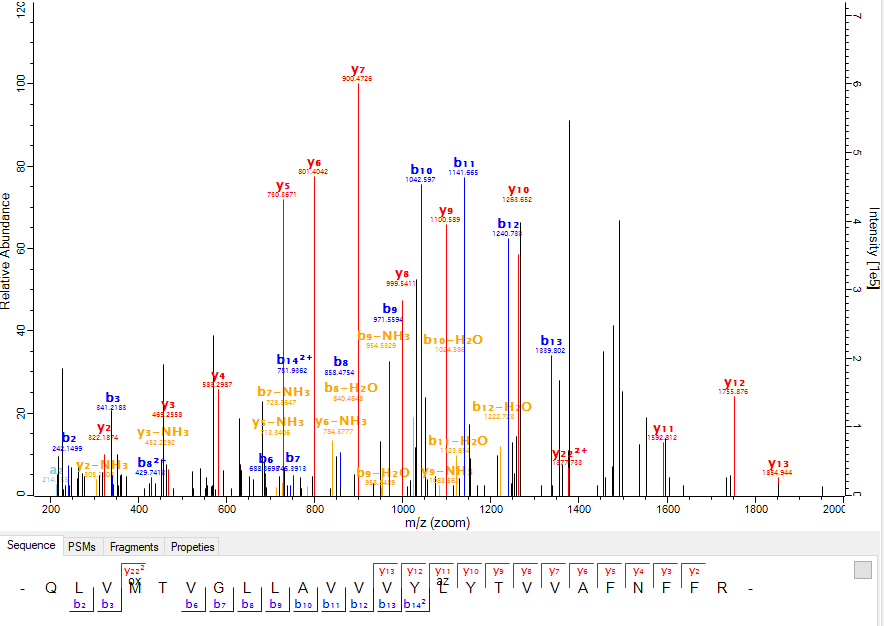


Figure S5n. LRPALGEC**L^3193^**AR-AziP*m*


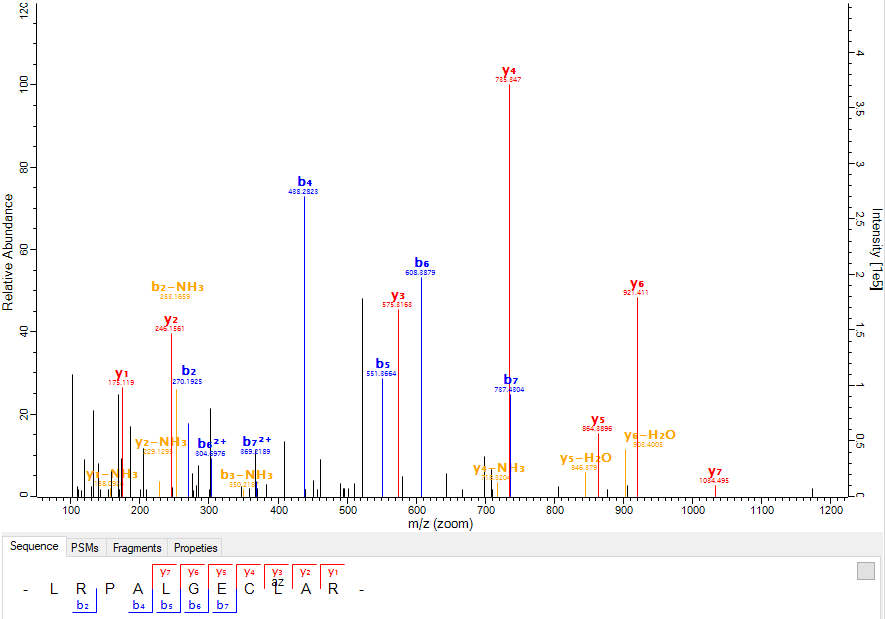


Figure S5o. DEFS**V^3402^**LCR-AziP*m*


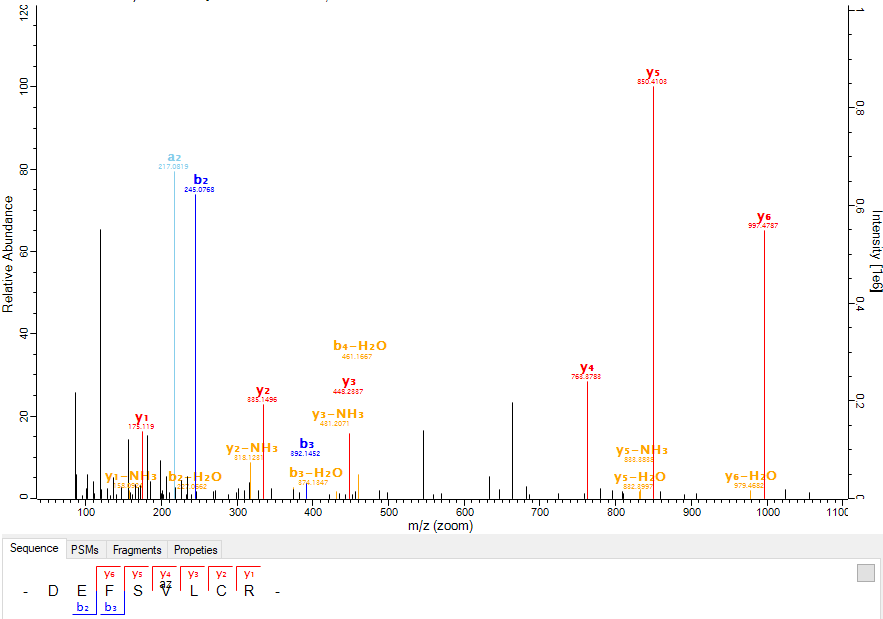


**Supplementary Figure S6. Mass spectra of AziPm photolabeled pig RyR1 peptides.** Residues detected with an AziP*m* photomodification are bolded, with residue ID in superscript. Colored intensities denote the identified peptide b and y ion fragments for the peptide sequence assignment. Identified a+ (light blue), b^+^ (red), b^2+^ (red), y^+^ (blue), and y^2+^ (blue) ions are labeled accordingly. Residues detected with an AziP*m* photomodification are labeled as “az”.

Figure S6a. VAHALCSH**V^1689^**DQAQLLHALEDAHLPGPLR-AziP*m*


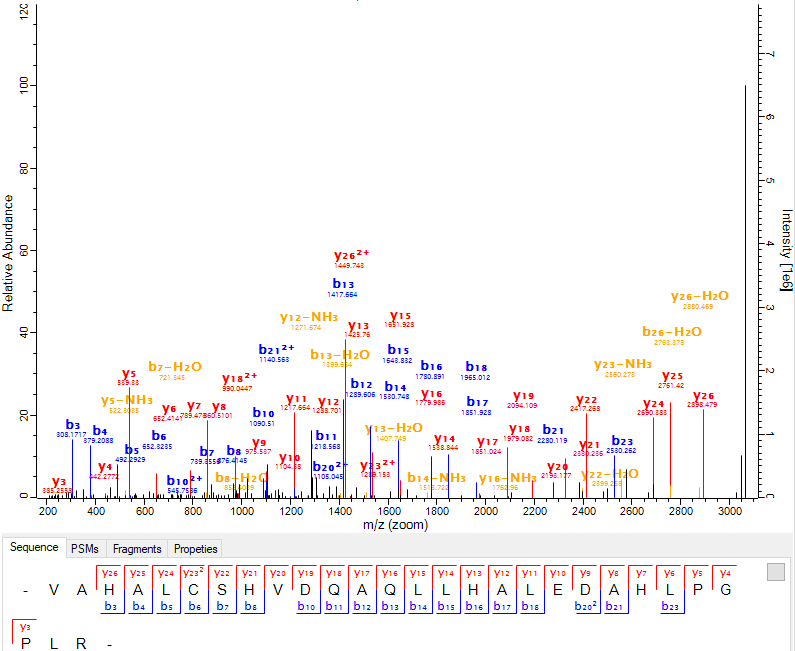


Figure S6b. LMSL**L^2068^**EK -AziP*m*


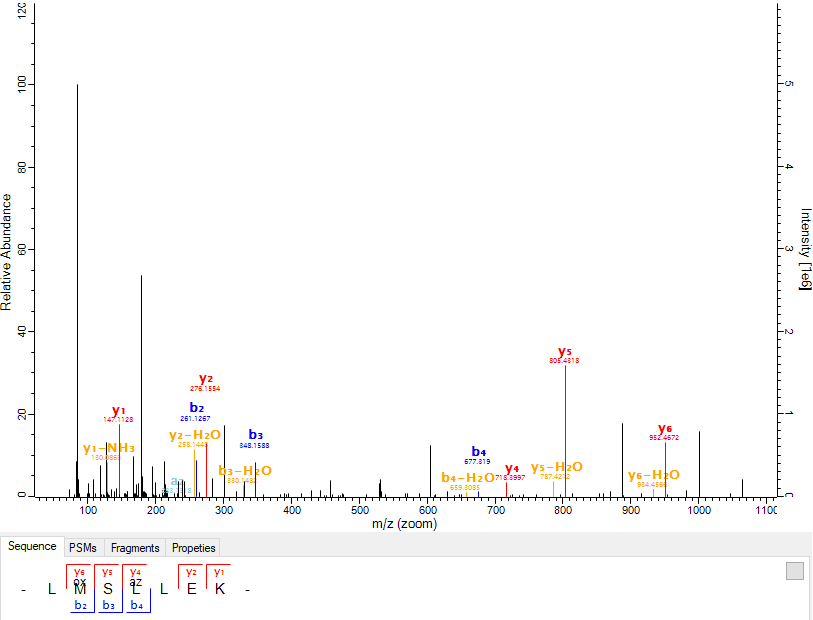


Figure S6c. SLLIVQMGPQEENLMIQS**I^2183^**GNIMNNK -AziP*m*


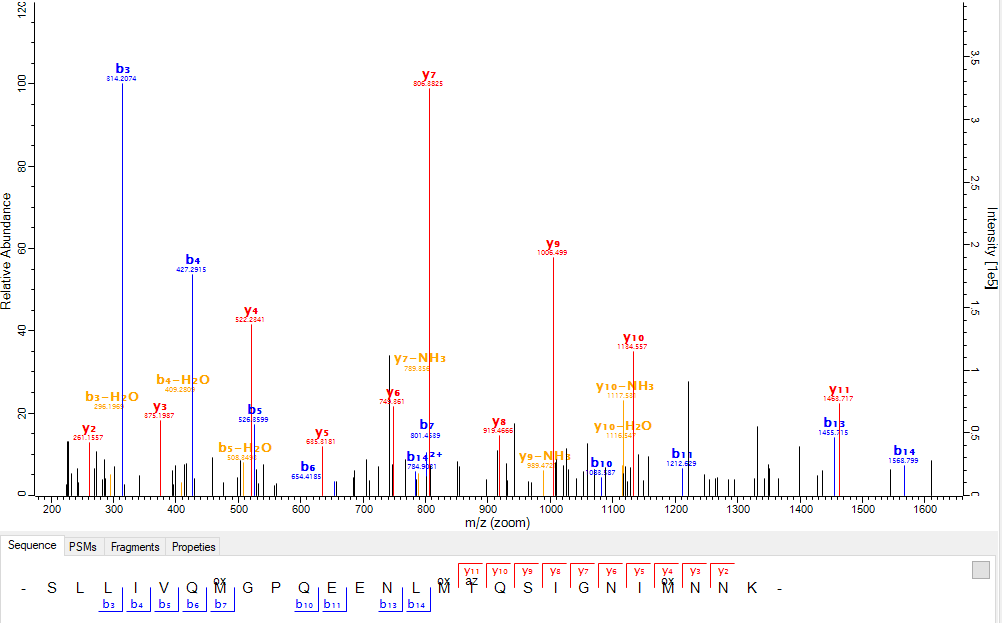


Figure S6d. YL**C^2555^**LAVLPLITK -AziP*m*


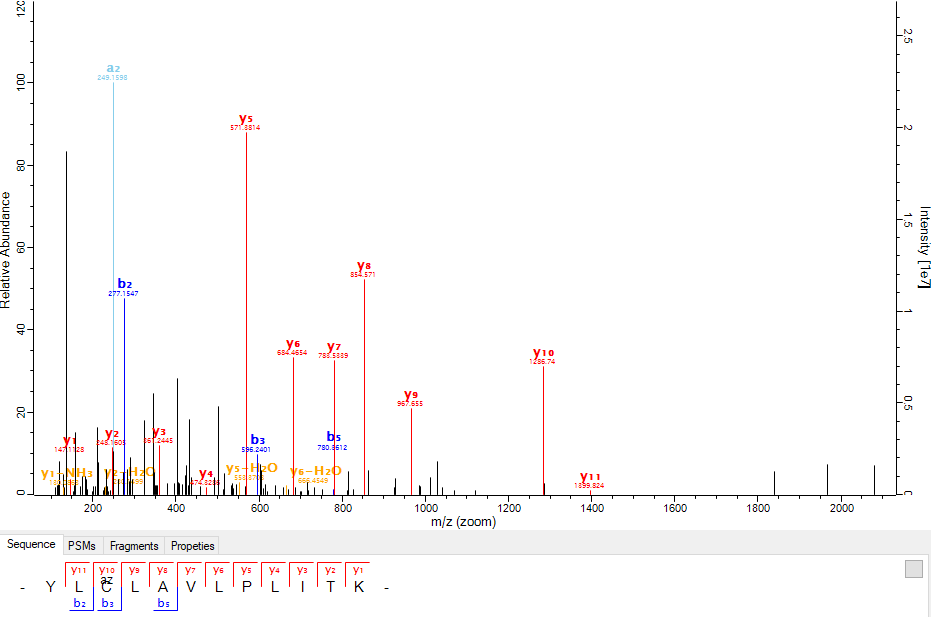


Figure S6e. **M^3634^**TPLYNLPTHR -AziP*m*


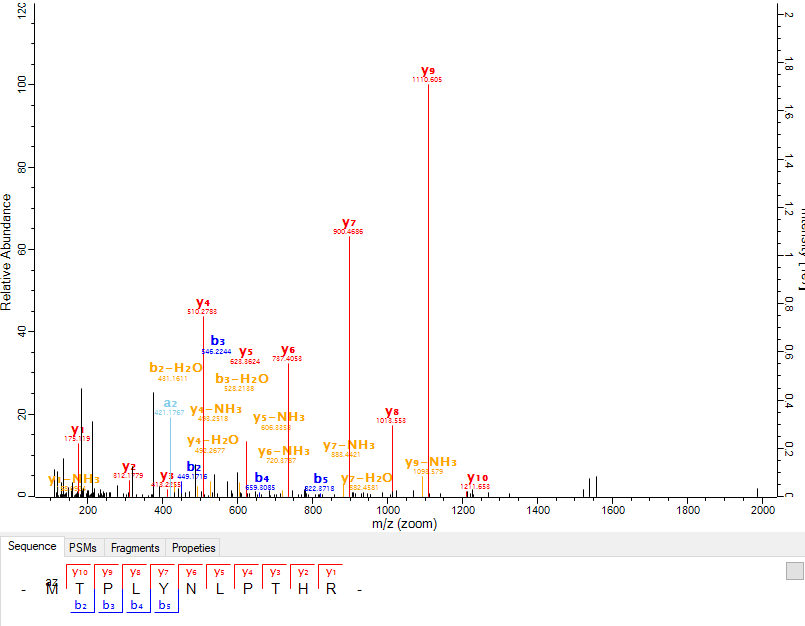


Figure S6f. GETGAMVSST**L^3793^**K -AziP*m*


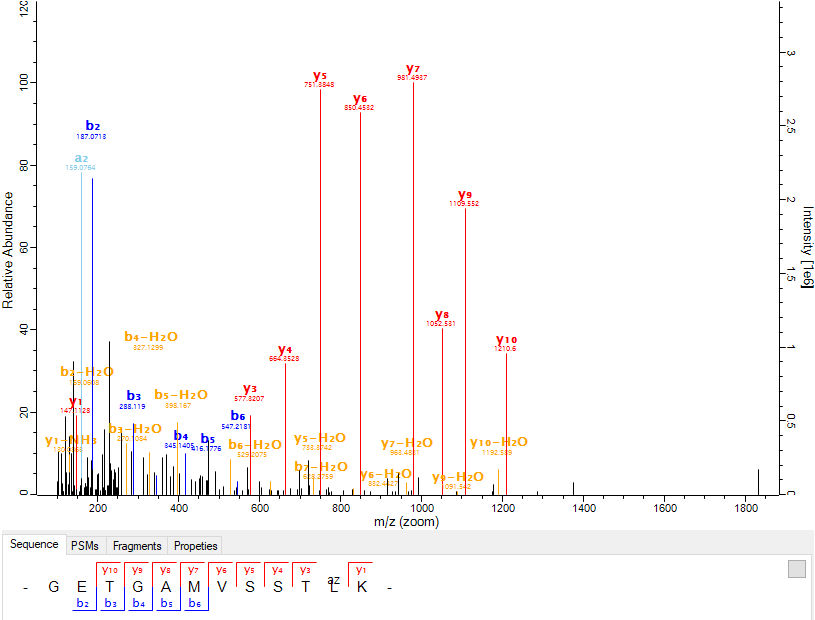


Figure S6g. QMVDMLVESSSNVEM**I^4053^**LK -AziP*m*


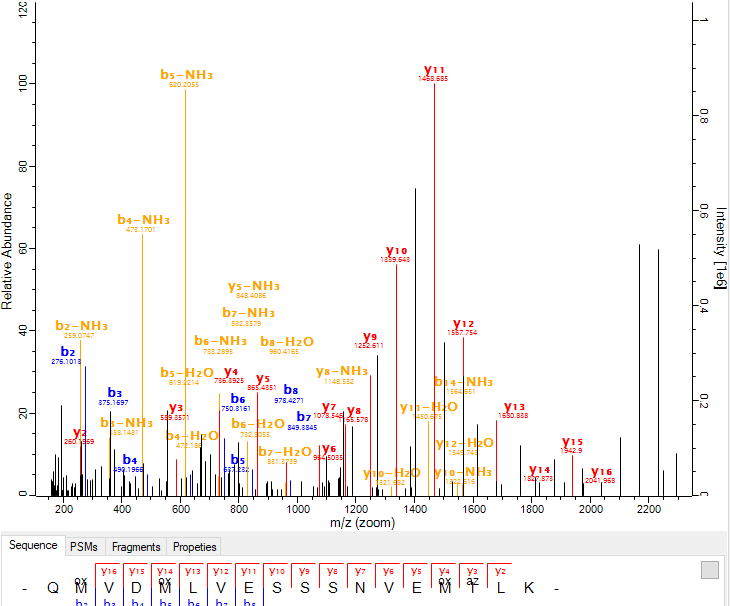


Figure S6h. RQF**I^4213^**FDVVNEGGESEK -AziP*m*


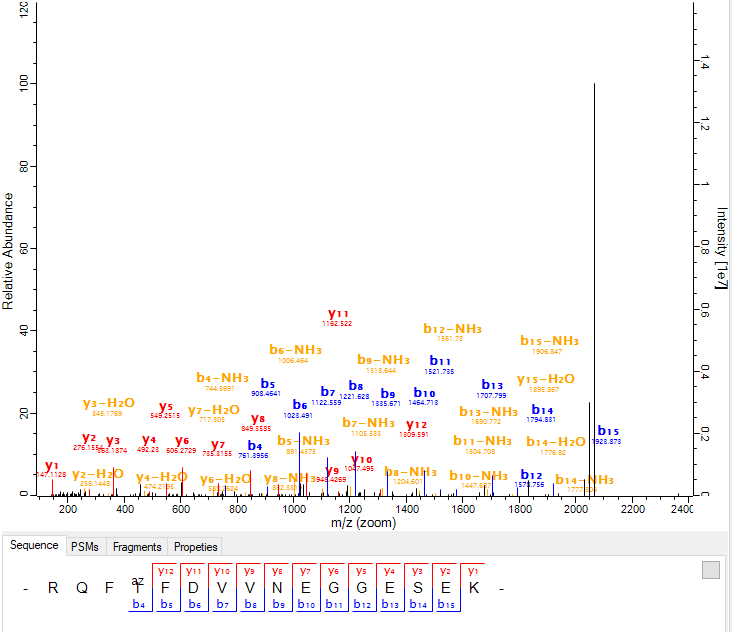


Figure S6i. FLNY**L^4553^**SR -AziP*m*


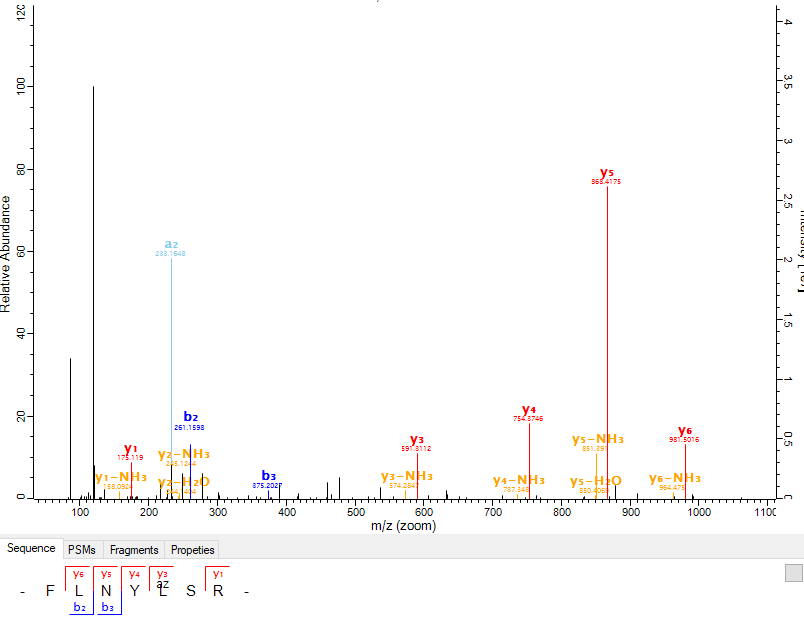


Figure S6j. NFYTLRFLALFL**A^4572^**FAINFILLFYK -AziP*m*


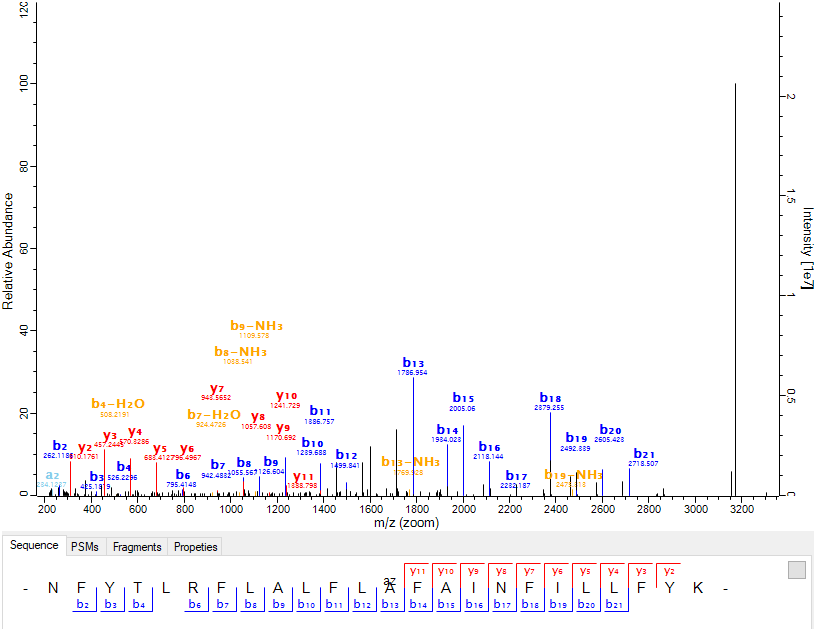


Figure S6k. LV**L^4713^**NTPSFPSN**Y**WDK-AziP*m*


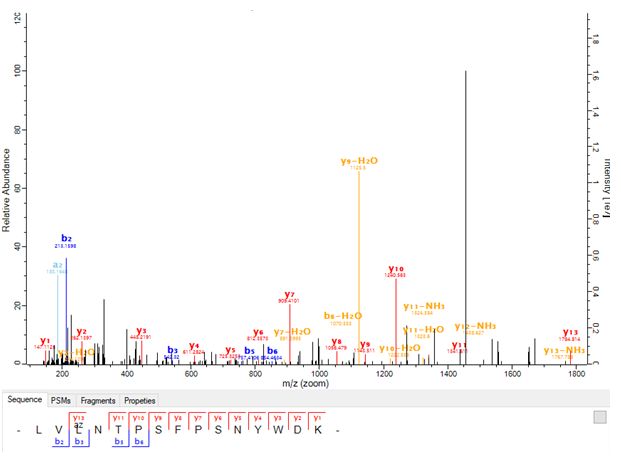


Figure S6l. IAEL**L^4735^**GMDLASLEITAHNER-AziP*m*


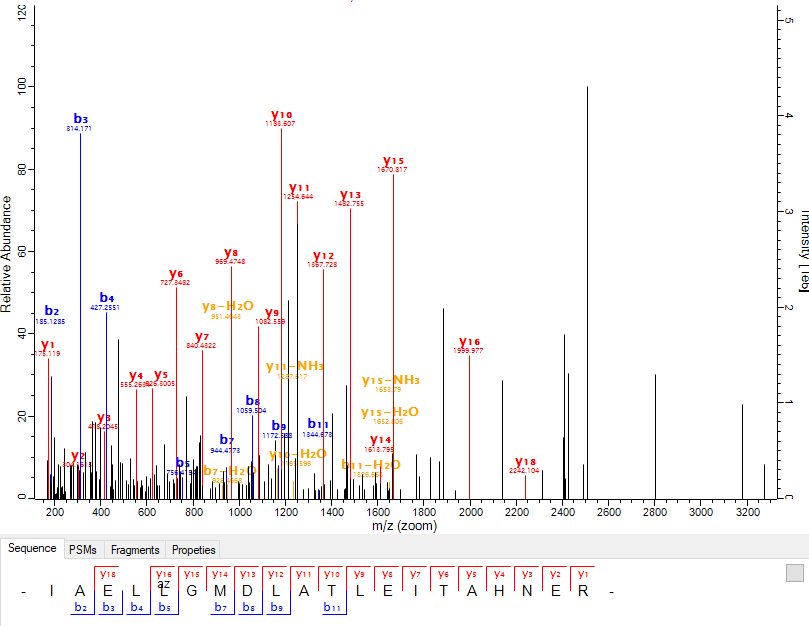


Figure S6m. TILSS**V^4828^**THNGK-AziP*m*


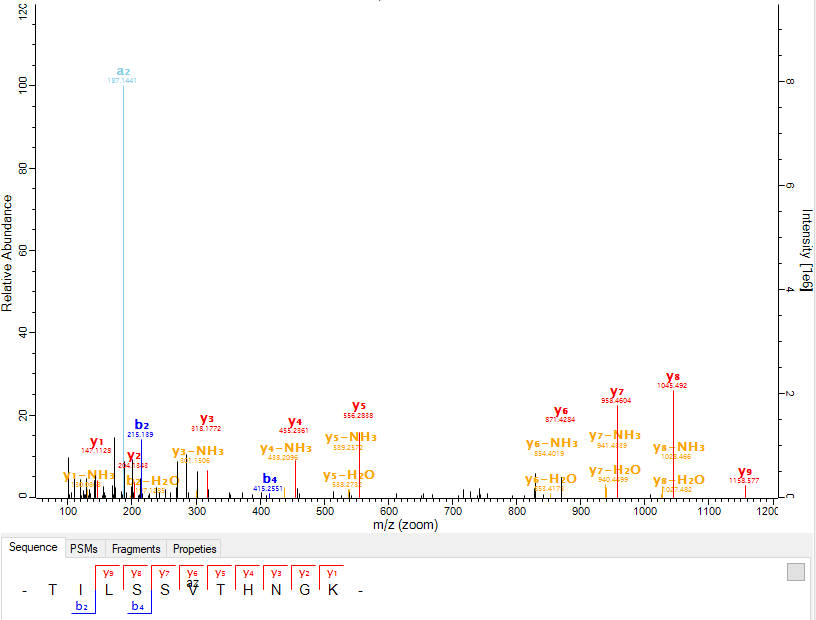


Figure S6n. QLVMTVGLLAVVVY**L^4848^**YTVVAFNFFR-AziP*m*


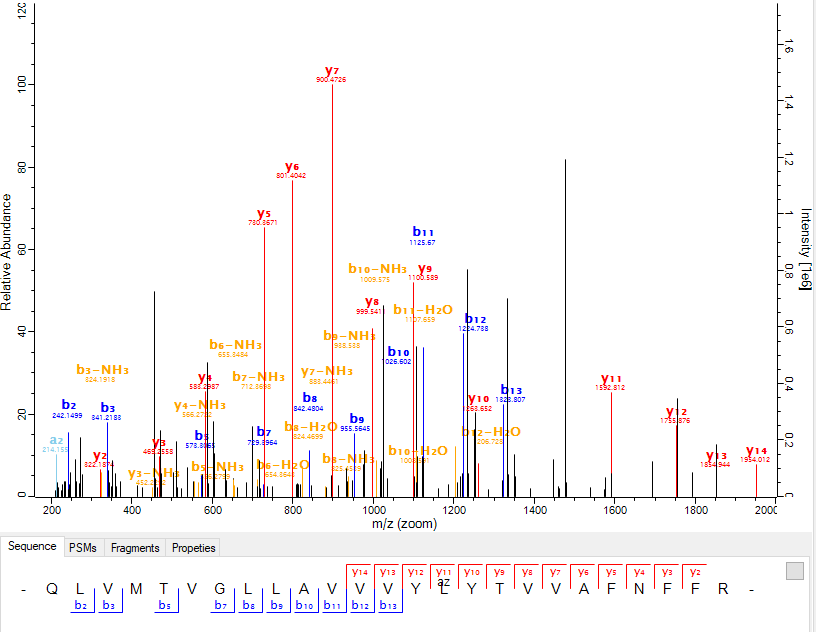


Figure S6o. AGGGIGDEIEDPAGDEYE**L^4909^**YR-AziP*m*


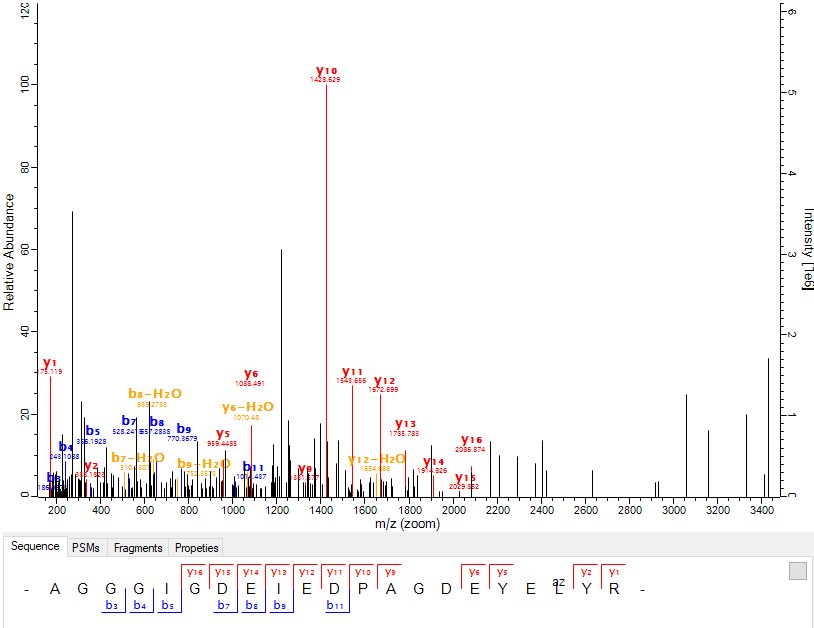


**Figure S7. Mass spectra of AziPm photolabeled Pig RyR1 R615C.** Residues detected with an AziPm photomodification are bolded, with residue ID in superscript. Colored intensities denote the identified peptide b and y ion fragments for the peptide sequence assignment. Identified a+ (light blue), b^+^ (red), b^2+^ (red), y^+^ (blue), and y^2+^ (blue) ions are labeled accordingly. Residues detected with an AziPm photomodification are labeled as “az”.

Figure S7a. VAHALCSH**V^1689^**DQAQLLHALEDAHLPGPLR-AziP*m*


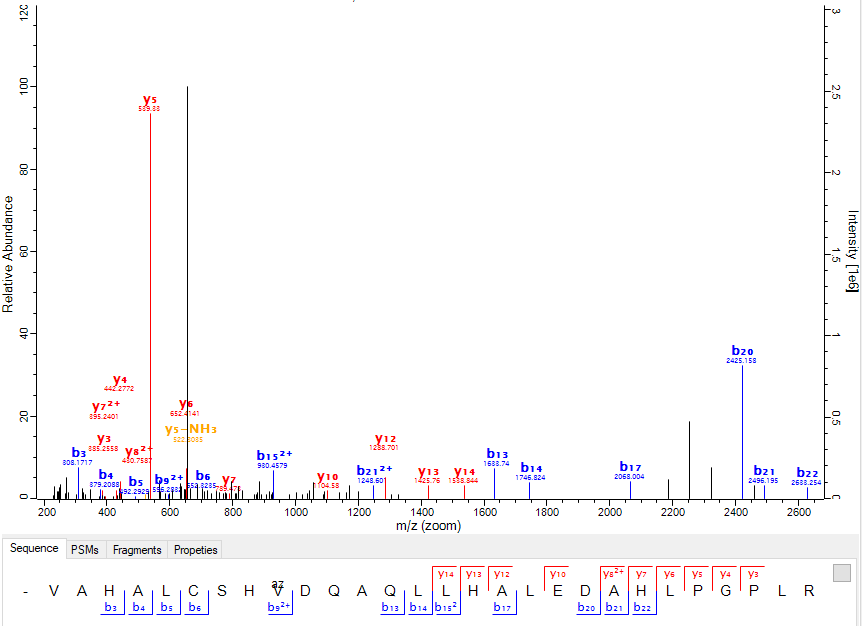


Figure S7b. LMSLLEKV**R^2072^**-AziP*m*


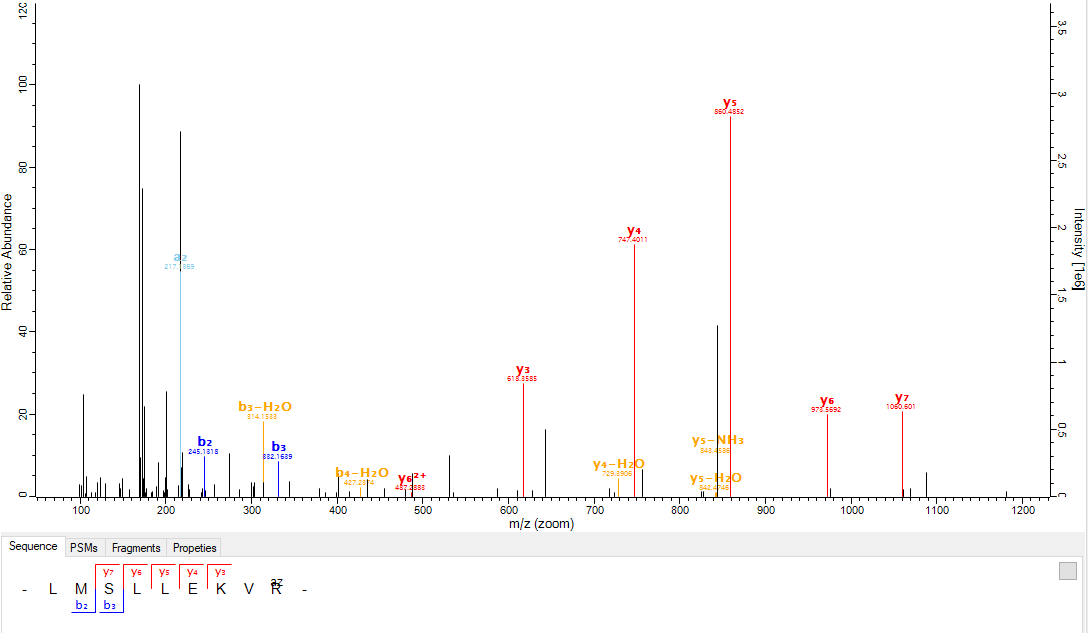


Figure S7c. SLLIVQMGPQEENLMIQS**I^2183^**GNIMNNK-AziP*m*


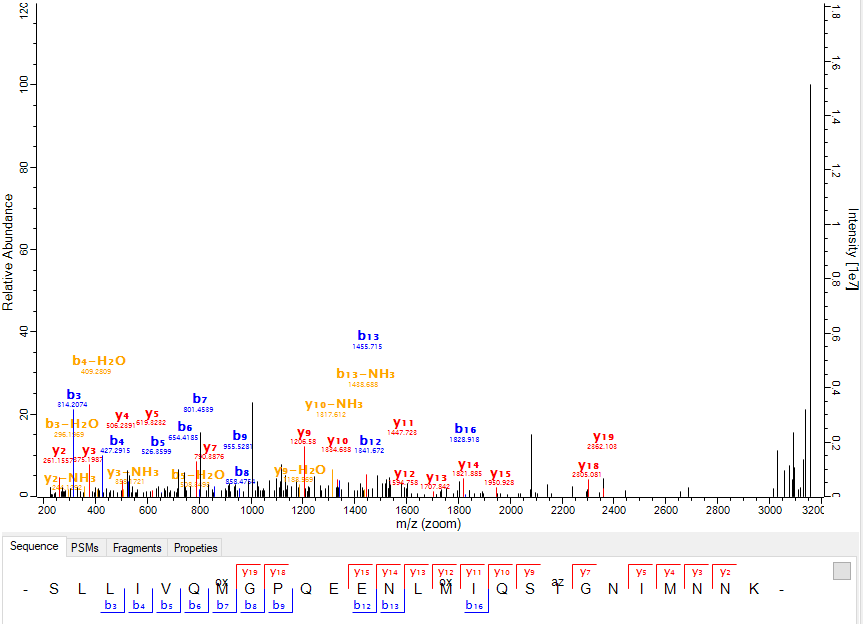


Figure S7d. **M^3634^**TPLYNLPTHR-AziP*m*


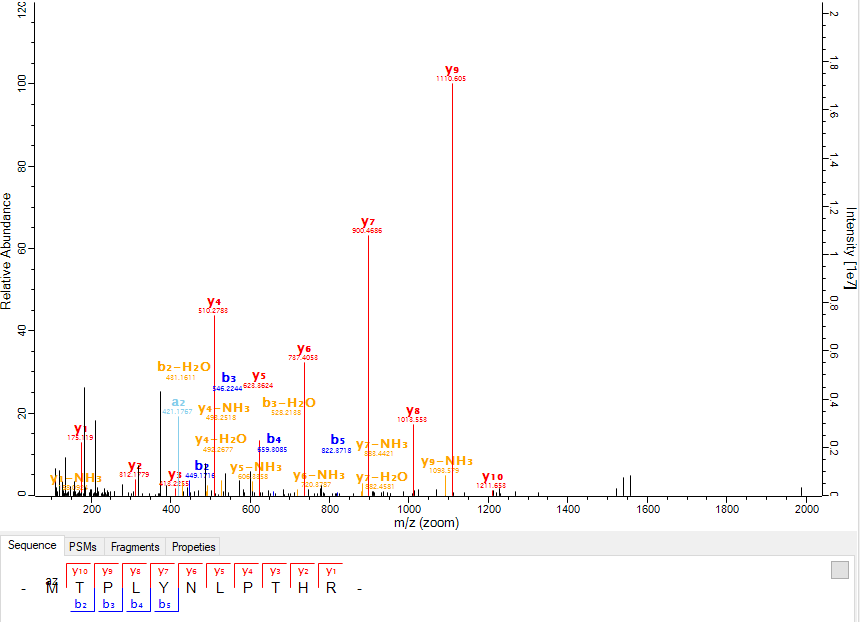


Figure S7e. GETGAMVSST**L^3793^**K-AziP*m*


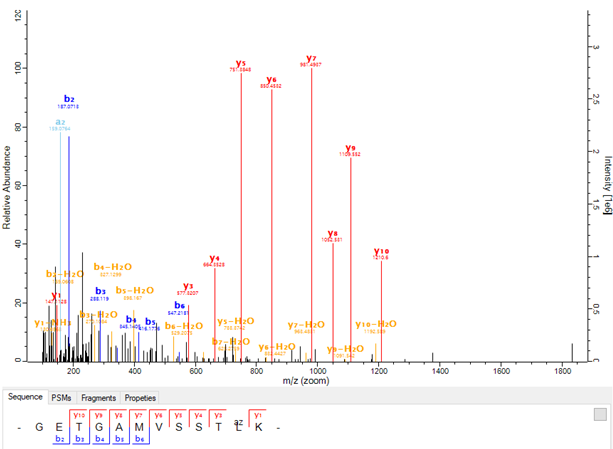


Figure S7f. QMVDMLVESSSNVEM**I^4053^**LK-AziP*m*


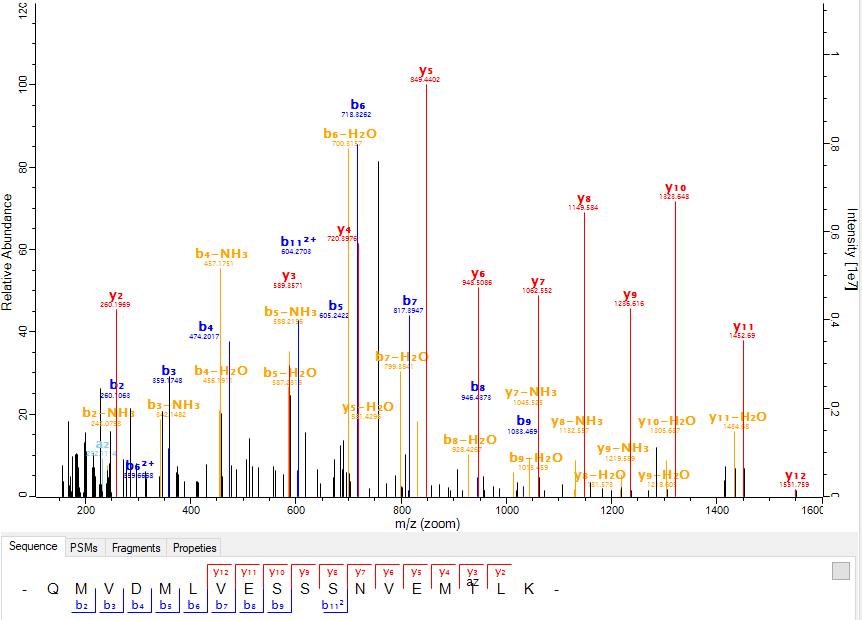


Figure S7g. RQF**I^4213^**FDVVNEGGESEK-AziP*m*


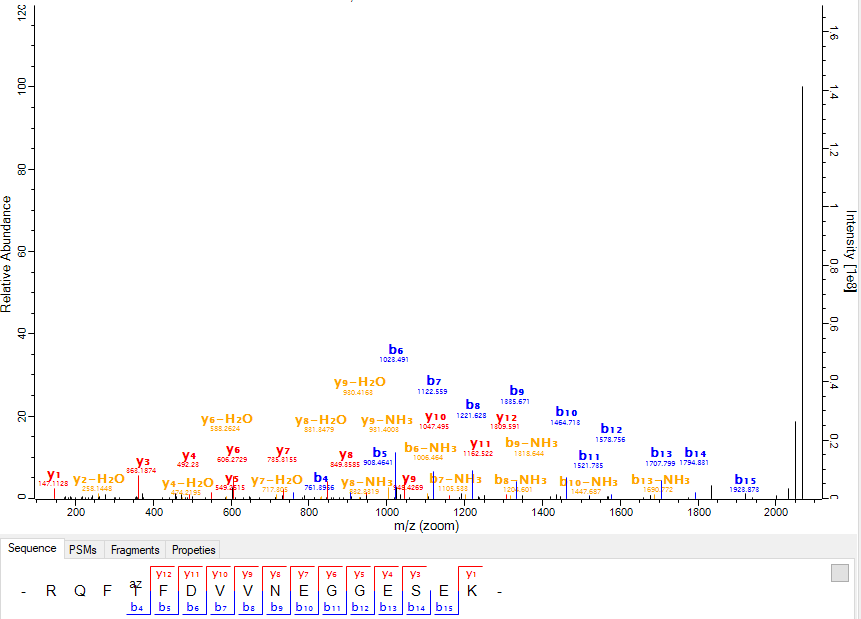


Figure S7h. FLNY**L^4553^**SR-AziP*m*


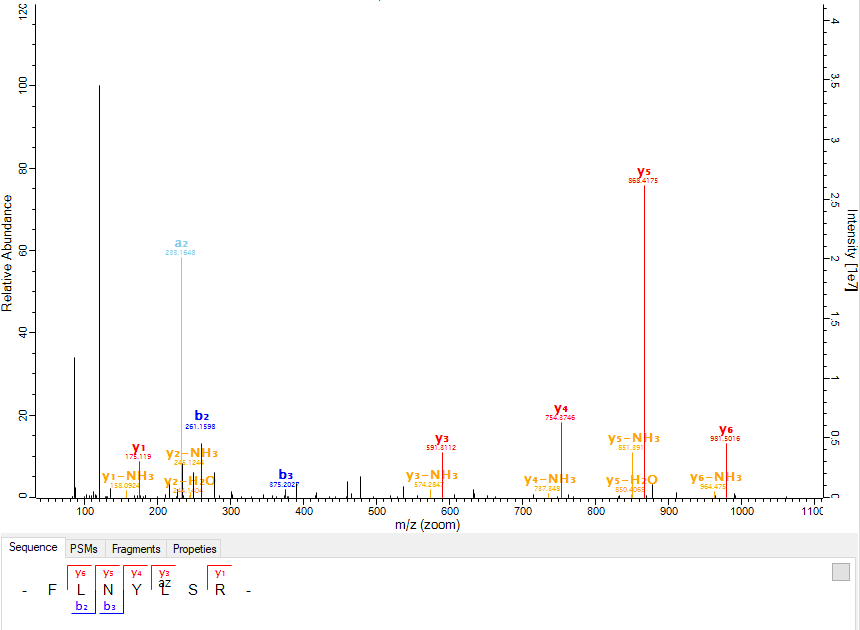


Figure S7i. NFYTLRFLALFL**A^4572^**FAINFILLFYK-AziP*m*


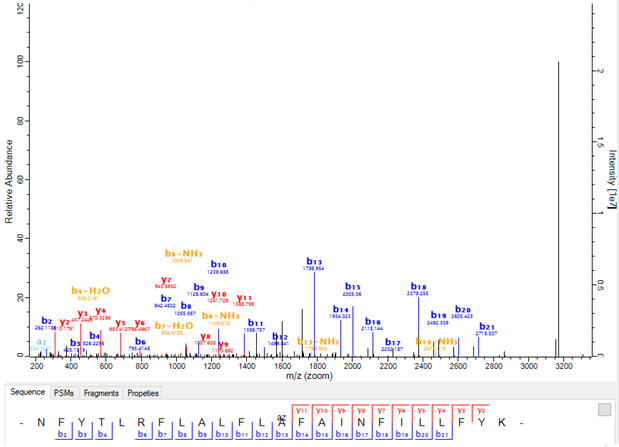


Figure S7j. LV**L^4713^**NTPSFPSNYWDK-AziP*m*


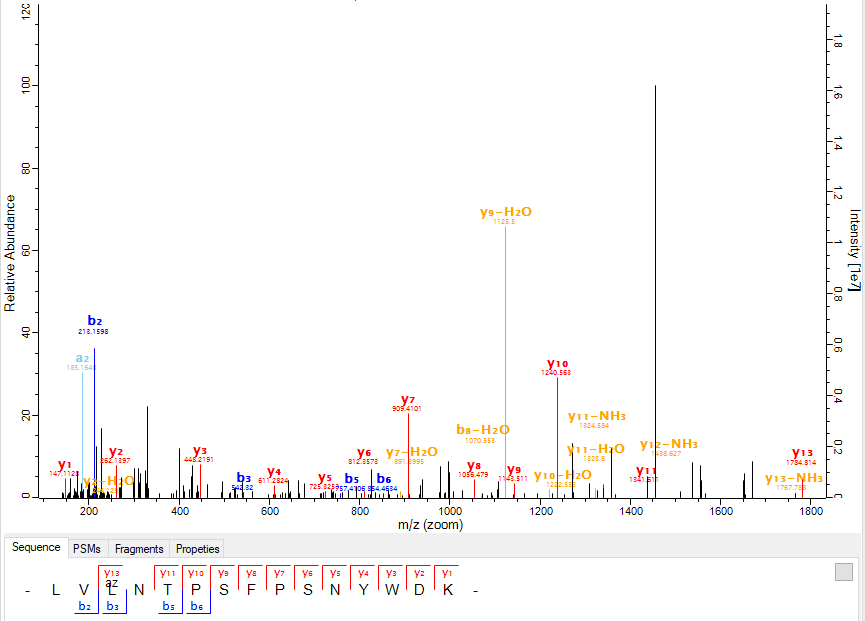


Figure S7k. IAEL**L^4735^**GMDLASLEITAHNER-AziP*m*


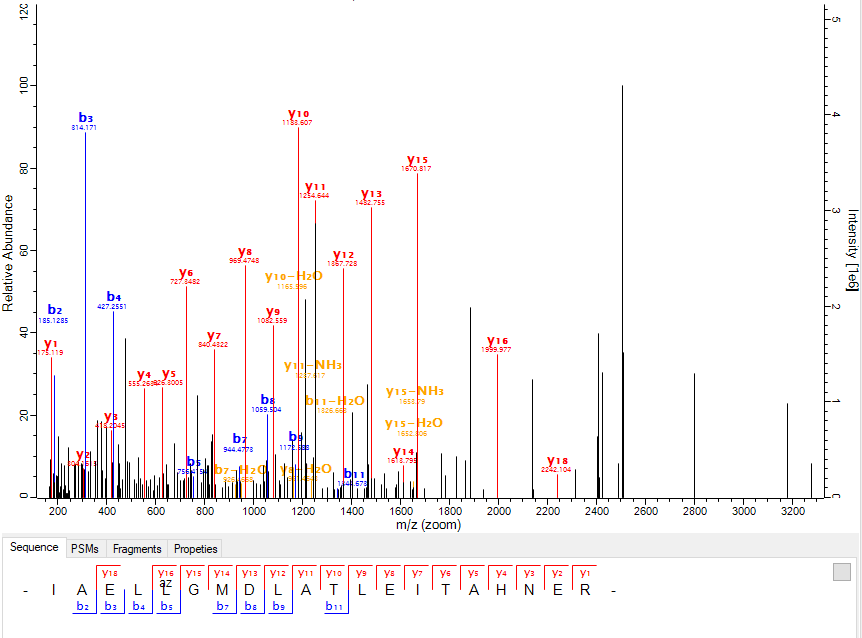


Figure S7l. TLRT**I^4824^**LSSVTHNGK-AziP*m*


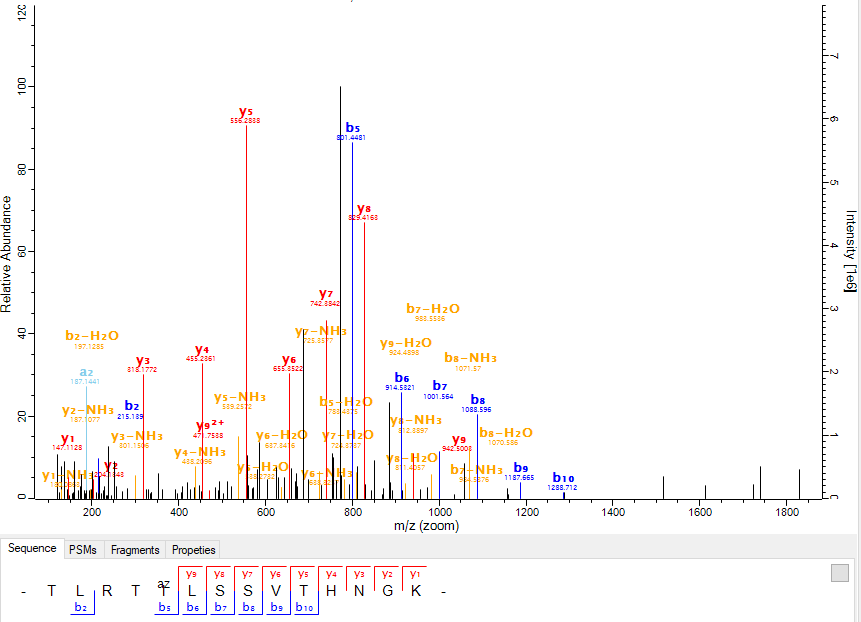


Figure S7m. TILSS**V^4828^**THNGK-AziP*m*


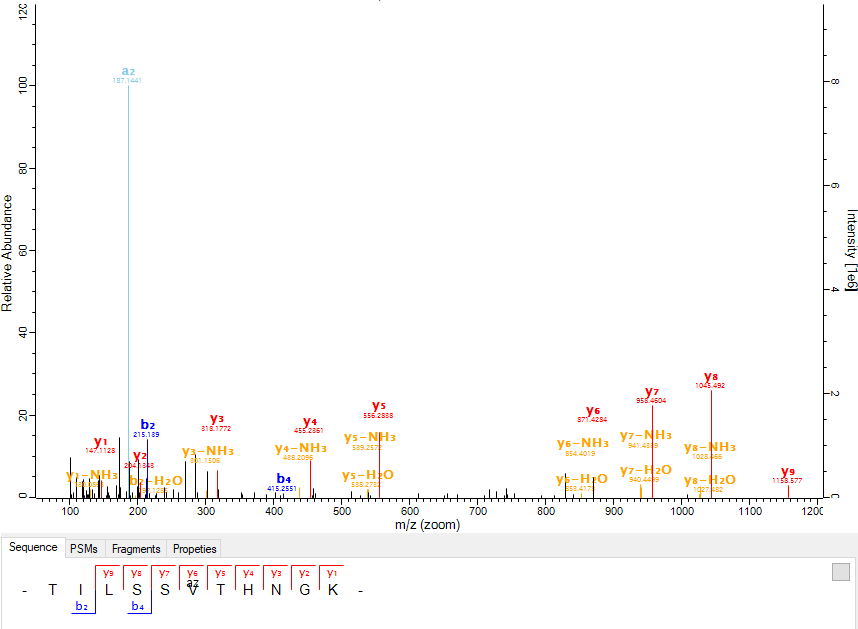


Figure S7n. QLVMTVGLLAVVVY**L^4848^**YTVVAFNFFR-AziP*m*


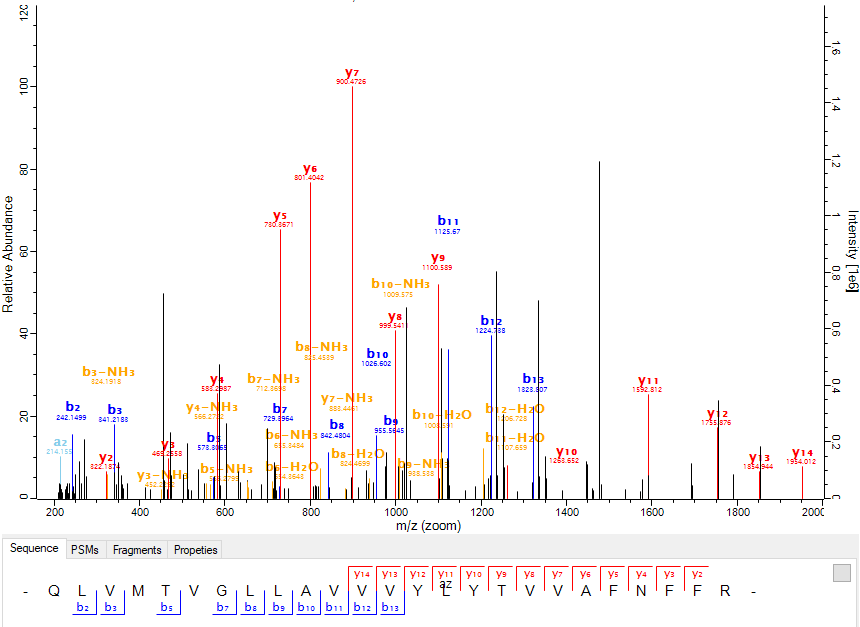


Figure S7o. AGGGIGDEIEDPAGDEYE**L^4909^**YR-AziP*m*


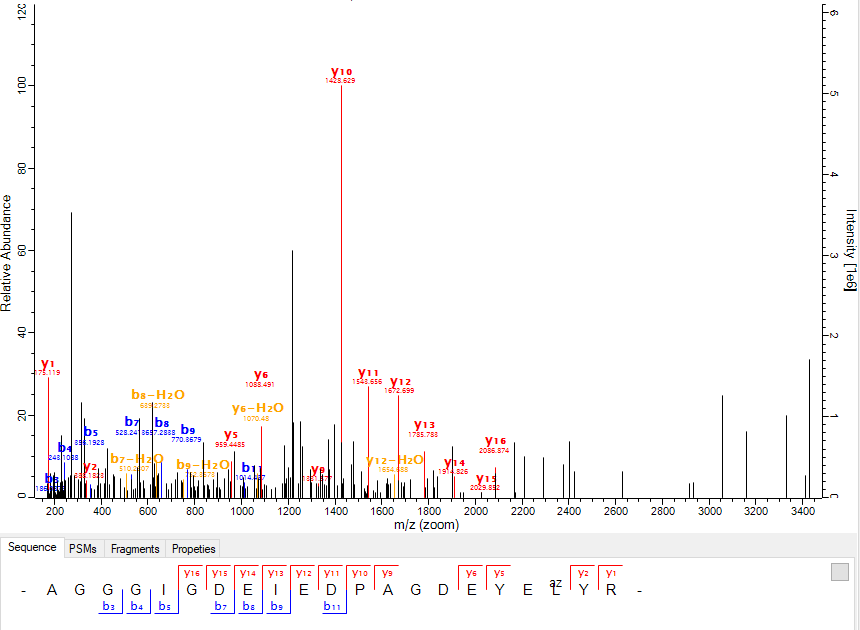


Figure S7p. **L^3189^**RPALGECLAR-AziP*m*


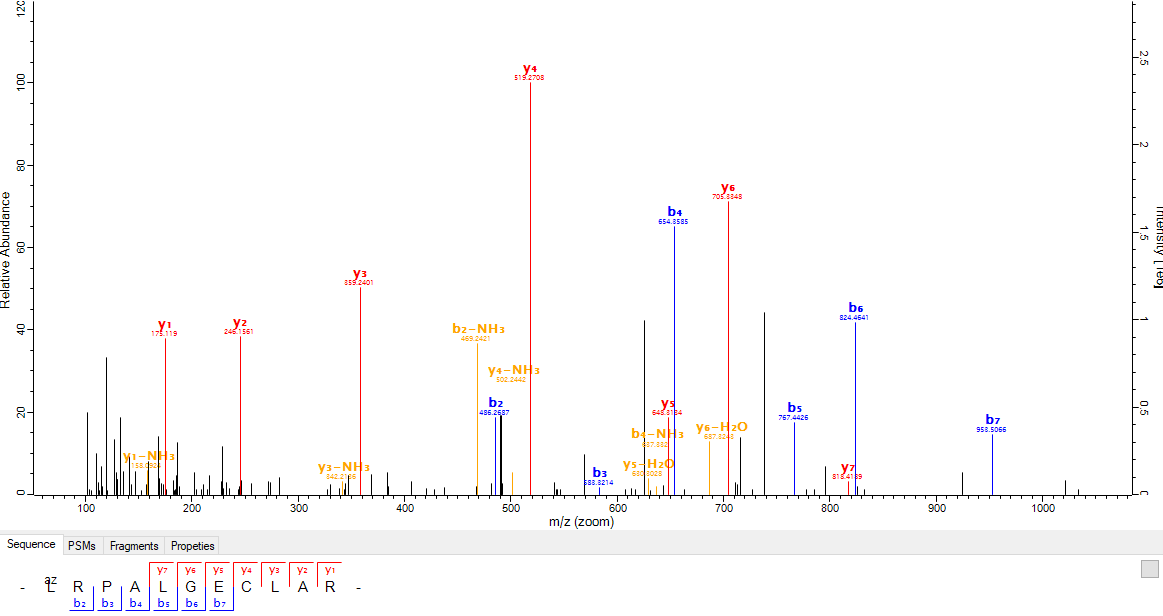


|  |
| --- |
| **Supplementary Figure S8.** Plots indicating various aspects of convergence of free energy MD simulations. For panels a through e, protein bound state decoupling on left and bulk aqueous phase decoupling on right.   1. Cumulative sum of ∆G for each window. 2. Per-window ∆G, not summed. 3. Discrepancy in ∆G for each window, between forward (λ increasing) and backward (λ decreasing) directions. A kernel density estimation (KDE) of the probability distribution of these values is shown on the right portion of this subpanel. Smaller values imply better convergence. 4. Discrepancy in ∆G between first and last half of samples for each window. Smaller values imply better convergence. 5. Convergence plot, depicting what fraction of simulation time (x-axis) is necessary to achieve a particular magnitude of discrepancy between forward and backward sampling (y-axis). 6. Cumulative and individual ∆G for the thermodynamic integration (TI) calculation to calculate the energetic cost of the DBC restraint 7. Titration curve, showing fraction of protein sites occupied as a function of propofol concentration |
