# Supplementary material for: Propofol binds and inhibits skeletal muscle ryanodine receptor 1
Source: Br J Anaesth. 2024 Sep 19;133(5):1093–100. doi: 10.1016/j.bja.2024.06.048 (PMC11488158; doi:10.1016/j.bja.2024.06.048)
Supplement: Multimedia component 2 [file mmc2.docx]

**Detailed Methods**

**[^3^H]Ryanodine binding assay.** The [^3^H]ryanodine binding assay was carried out as previously described (1). Heavy SR (HSR) vesicles were provided by Dr. Francisco Alvarado (Cardiovascular Research Center, School of Medicine and Public Health, University of Wisconsin, Madison, WI). The binding assays were performed at fixed [Ca^2+^] = 100 nM (pCa 7) or 10 μM (pCa 5). Wild-type HSR vesicles were the only HSR vesicles available.

Briefly, propofol (1–100μM) or AziP*m* (1–48 μM) was added to a binding mixture with 100 μl volume, containing 50μg of HSR protein, 200 mM KCl, 100 mM HEPES buffer (pH 7.2), 5 nM [^3^H]ryanodine (56 Ci/mmol, PerkinElmer, #NET950) , 1 mM EGTA and enough CaCl_2_ to set free [Ca^2+^] at 100 nM (pCa 7) or 10 µM (pCa 5). The Ca^2+^/EGTA ratio for these solutions was calculated using MaxChelator (WEBMAXC Lite V1.15; temperature=37 ^o^C, pH 7.2, Ionic=0.3N). Non-specific binding was determined under the same conditions in the presence of 20 μM unlabeled ryanodine (Millipore-Sigma, #559276).

Binding reactions were incubated for 2 hours at 37°C. Prior to the reaction, the filters were presoaked with 0.5% polyethylenimine for 30 mins. Then the reaction was stopped by vacuum filtration and washed three times with distilled water. The filters were soaked in 10 mL Ecolite(+)^TM^ scintillation cocktail overnight. [^3^H]Ryanodine binding was measured by liquid scintillation in a PerkinElmer Tri-Carb 2800TR counter. Experiments were done in triplicate.

**Purification of rabbit or pig RyR1 from rabbit/pig muscle.** Approximately 200 g of frozen rabbit/pig skeletal muscle was blended for 120 s in 2 × 500 ml of 10 mM tris/maleate (pH 6.8), 10% sucrose, 1 mM dithiothreitol (DTT), 1 mM EDTA, 200 μM PMSF, and 1 mM benzamidine. The mixture was centrifuged at 4°C for 10 min at 7000g. The supernatant was filtered through a cheesecloth and centrifuged at 4°C for 40 min at 40,000g. Pellets were solubilized in buffer S1 containing 20 mM Hepes (pH 7.4), 1 M KCl, 2 mM TCEP, 150 μM PMSF, 1 mM EGTA, 1% CHAPS, and 0.2% soybean phosphatidylcholine with 100 μl of protease inhibitor cocktail (Protease Inhibitor Cocktail Set III, EDTA-Free, Calbiochem). After stirring for 1 hour at 4°C, the solubilized membranes were diluted with 120 ml of buffer S2 (as for buffer S1 but lacking 1 M KCl). His-GST-FKBP12.6 (~5 mg) was then added to the solubilized membranes, with incubation for 2 hours at 4°C. After ultracentrifugation for 45 min at 200,000g, the supernatant was filtered and mixed with 2 to 3 ml of GS4B resin (Cytiva) pre-equilibrated with buffer S3 containing 20 mM HEPES (pH 7.5), 0.5 M KCl, 0.5% CHAPS, 0.2% soybean phosphatidylcholine, 1 mM EGTA, 2 mM TCEP, 150 μM PMSF, and 100 μl of protease inhibitor cocktail, with stirring for 3 hours at 4°C. The mixture was poured into a column and washed with 10-CV buffer S3. Next, 10 ml of buffer S4 (identical to S3 except with 75 mM HEPES) together with 1.5 mg of TEV protease was added to the column and incubated overnight to elute RyR1 from the column. The eluents were concentrated to 500 μl, applied to a gel-filtration column (Superose 6 10/300 GL, Cytiva) and eluted with buffer S5 containing 15 mM Tris (pH 7.5), 0.15 M NaCl, 0.5% CHAPS, 0.001% 18:1 (Δ9-cis) phosphatidylcholine (DOPC), 2 mM TCEP, and 1 mM EGTA. Peak fractions containing RyR1 complexes were combined and concentrated to ~ 2 mg/ml. Concentration was estimated using NanoDrop [absorbance at 280 nm, 1% (w/v) = 1.0].

**RyR1 proteolipsome reconstitution.** RyR1 was reconstituted into proteoliposomes as previously described (2). Briefly, a 5:3 mixture of 1,2-dioleoyl-sn-glycero-3- phosphoethanolamine (DOPE) and 1,2-dioleoyl-sn-glycero-3- phosphocholine (DOPC) (Avanti Polar Lipids) were dried into a thin film, followed by overnight incubation in a vacuum chamber. Dried lipids (1 mg) were solubilized with 400 μl of rabbit RyR1 (0.7 mg/ml) in buffer S5, and the mixture was dialyzed overnight using a 3.5-kDa membrane in 10 mM HEPES (pH 7.4), 500 mM NaCl, 0.1 mM EGTA, 0.2 mM CaCl_2_, 0.15 mM PMSF, and 1 mM DTT. Following dialysis, the samples were aliquoted, flash-frozen in liquid nitrogen, and stored at −80°C for later use.

**Planar lipid bilayer methods: single channel recordings.** For all experiments, planar lipid bilayers were formed from a mixture of DOPE:DOPC= 5:3 (mol/mol) dissolved in decane with a final lipid concentration of 20 mg/mL. For single channel recordings, an integrated chip-based recording setup Orbit mini and EDR2 software (Nanion Technologies, Livingston, NJ) was employed. Recordings were obtained in parallel with multielectrode-cavity-array chips (Ionera Technologies, Freiburg, Germany). The cis and trans chambers contained symmetrical solutions of 250 mM HEPES, 150 mM KCl, 1 mM EGTA (pH 7.3), 0.2 mM CaCl_2_ ([Ca^2+^] free = 0.1 μM). The free concentration of Ca^2+^ was calculated with WebMaxC program (v.2.50; https://somapp.ucdmc.ucdavis.edu/pharmacology/bers/maxchelator/webmaxc/webmaxcS.htm). Ca^2+^ concentrations were further verified using PerfectIon™ Combination Calcium Electrode unit (Mettler Toledo). To promote fusion to prepared suspended bilayers, 5% glycerol was incorporated into the proteoliposomes. In brief, prepared proteoliposomes were mixed 1:1 in 250 mM HEPES (pH 7.3), 150mM KCl, and 10% glycerol. The samples were then freeze-thawed and sonicated 3-5 times to incorporate the glycerol into the proteoliposome lumen. 1–2 μL RyR1 proteoliposomes were added to the cis chamber. To further promote fusion the voltage was maintained at +40 mV. Recordings were started at the point of successful RyR1 insertion. Propofol was introduced by the displacement of 3–30% of the cis chamber volume with recording solution containing 60 μM or 100 μM propofol. Concentrations of propofol aqueous stocks were determined by measuring the absorbance at 270 nm, using ε 1584 M^-1^ cm^-1^ (3). All RyR1 measurements were conducted at 22°C and at a constant voltage of –60mV. Recordings were filtered at final bandwidth of 10,000Hz. Clampfit software (10.6, Molecular Devices, San Jose, CA) was used to analyze current traces and only channels with a conductance greater then 700 pS were included in the analysis (4)

**Calcium imaging in human skeletal myotubes.** Human skeletal muscle cells (HSMC, Sigma, #150-05F) were maintained in growth medium (Sigma, #151-500) in a 5% CO_2_ atmosphere at 37°C. The medium was changed every other day. Cells were passaged when they reached 80-90% confluence.

To induce differentiation, the cells were plated at 20,000 per cm^2^ on 35 mm dishes (World Precision Instruments, Inc, #FD 35-100) pre-coated with collagen I (Sigma-Aldrich, Louis, MO, USA) at 37 °C and 5% CO2. The cells were incubated overnight in growth medium, then the growth medium was replaced with differentiation medium (Sigma, #151D-250). The differentiation medium was changed every other day. Multinuclear myotubes typically formed within 5-6 days.

**Calcium fluorescence measurements.** Differentiated myotubes were washed with the running HBSS buffer (Hepes-buffered salt solution (HBSS, Sigma, #H-4891) containing 1.8 mM CaCl_2_, 0.8 mM MgCl_2_). The cells were loaded with 1.5 μM Fura-2 AM (Invitrogen, #F1221) and 20% BSA in the running HBSS buffer for 45 min. The cell was washed again and incubated with 150 µL running buffer for 15 min to allow de-esterification of the acetoxymethyl ester from the now-intracellular Fura-2 AM. The cells were then ready to be excited alternately at 340 nm and 380 nm. The fluorescent maker Fura-2 binds to intracellular Ca^2+^, with the ratio of emission due to excitation at 340 nm and 380 nm directly related to the concentration of Ca^2+^. Hence release of calcium stores in the SR into the cytoplasm due to RyR1 channel opening would be expected to result in an increased 340:380 nm ratio (5–7). The ratio of 340nm:380nm was measured using a fluorescent microscope (Olympus IX-70, Japan) equipped with a cooled high-speed digital video camera (Hamamatsu, Japan), and using the software MetaFluor for Olympus (version 7.10.4.407, MetaMorph 2020, Molecular Devices, LLC). 40-50 cells were usually chosen to measure the changes in Fura-2 fluorescence.

*Experiment 1: Reactivity to ryanodine.* Running HBSS buffer containing increasing concentrations of ryanodine (2, 5, 10, 25, 50, 100, 200, 500, 1000 nM; in Figure 3 plotted on a logarithmic scale) were added to the myotubes. Changes in Fura-2 340/380nM ratios were measured.

*Experiment 2: Reactivity to propofol with 1 µM ryanodine.* From the dose response curve for ryanodine we found that 1 µM ryanodine resulted in the maximum Fura-2 340:380 nm ratio, and as a result this concentration was used to determine the inhibition, if any, caused by different concentrations of propofol. The various propofol concentrations (2, 5, 10, 20, 50, 100, 200, 300 µM) was added to 1µM ryanodine. The 340/380 nm ratios were measured using the same method in Experiment 1.

*Data analysis.* To trace the concentration-response curves, the data were normalized to the maximal response of cells. Then, the IC_50_ was calculated from the concentration response curves and all data were analyzed using PRISM 5.0 software (GraphPad Software, San Diego, CA, USA).

**Photolabeling of RyR1-FKBP12.6.** A final concentration of 5μM AziPm was added (with or without 200 μM propofol) to the purified RyR1-FKBP12.6 to a final protein concentration of 1 μg/μl. The 200 μM propofol was added to test for labeling protection (competition) in order to evaluate AziP*m* binding specificity. The samples were equilibrated on ice in the dark for 5 min and then irradiated for 30 min at 350 nm with an RPR-3000 Rayonet lamp in 1-mm path length quartz cuvettes through a 295-nm glass filter (Newport Corporation).

**In-solution protein digestion.** After UV exposure proteins were precipitated overnight at -20° C in 4 volumes of chilled acetone.  Protein was pelleted for 20 min at 16,000 x g at 4 °C then gently washed twice with 300 μL of chilled acetone. Protein pellets were air-dried before resuspension in 50 μl of 50 mM Tris-HCl, pH 8.0, 1% Triton X-100, and 0.5% SDS. Insoluble debris was pelleted by centrifugation at 16000x g. The samples were resuspension in final concentration of 50 mM NH_4_HCO_3_. Following 1 μL 0.5 M dithiothreitol (DTT) was added and samples were incubated at 56 °C for 30 min 0.55 M iodoacetamide (IAA) was then added and protein samples were incubated at room temperature in the dark for 45 min. Sequencing grade-modified trypsin (Promega) was added to a final 1:20 protease: protein ratio (w:w) with additional of 0.2% (w/v%) ProteaseMax™ surfactant. Proteins were digested overnight at 37° C. Trypsin digested peptides were diluted to 200 μL with final concentration of 100 mM NH_4_HCO_3_ and 0.02% ProteaseMAX Surfactant prior to the addition of sequencing grade chymotrypsin (Promega) to a final 1:20 protease:protein ratio (w:w). Proteins were digested overnight at 37° C. Acetic acid (AcOH) was added to until the pH < 2 and the peptide digests were incubated at room temperature for 10 min prior to centrifugation at 16000x g for 20 min to remove insoluble debris. The sample was desalted using C18 stage tips prepared in house. Samples were dried by speedvac and resuspended in 0.1% formic acid immediately prior to mass spectrometry analysis.

**In-gel protein digestion.** Photolabeled proteins were separated by SDS-PAGE. The identified band corresponding to rRyR1 was excised. Excised bands were distained, dehydrated and dried by speedvac before proteins were reduced by incubation at 56° C for 30 min in 5 mM DTT and 50 mM NH_4_HCO_3_. The DTT solution was removed and proteins were then alkylated by the addition of 55 mM IAA in 50 mM NH_4_HCO_3_ and incubation at room temperature for 45 min in the dark. Bands were dehydrated and dried by speed vac before resuspension in 100 μL 0.2% ProteaseMAX™ surfactant (Promega) and 50mM NH_4_HCO_3_ solution containing trypsin (sequencing grade, Promega) at a 1:20 protease:protein ratio (w:w). Proteins were digested for 12–16 hrs at 37°C. After trypsin digestion, the samples were diluted to 200 μL with final concentration of 100 mM NH_4_HCO_3_ and 0.02% ProteaseMAX™ Surfactant. The samples were further digested overnight at 37 °C with the addition of sequencing grade chymotrypsin (Promega) to a final protease:protein (w:w) ratio of 1:20. Multiple peptide extractions were performed in order to increase hydrophobic peptide retrieval from the gel. The peptide extractions were pooled from the different ratio of acetylnitrile and acetic acid. The final extractions were sonicated for 20 min and dried by speed vac before resuspension in 0.5% acetic acid and further acidified until the pH < 2. Samples were sonicated for 10 min prior to centrifugation at 16 000 x g for 20 min to remove insoluble debris. Samples were desalted using C18 stage tips prepared in house. Samples were dried by speed-vac and resuspended in 0.1% formic acid immediately prior to mass spectrometry analysis.

**Mass spectrometry.** Mass spectrometry was performed similarly to the previously reported procedure (8). Briefly, desalted peptides were injected into a Thermo LTQ Orbitrap XL Mass Spectrometer (Thermo Fisher Scientific, Waltham, MA, USA) or an Orbitrap Elite™ Hybrid Ion Trap mass spectrometer. Peptides were eluted with 100 min with linear gradients of ACN in 0.1% formic acid in water (v/v%) starting from 2% to 40% (85 min), then 40% to 85% (5 min) and finally 85% (10 min).

Spectral analysis was conducted using MaxQuant (9) to search b and y ions against the sequence containing rRyR1. Search parameters were 1 amu parent ion tolerance, 1 amu fragment ion tolerance, full tryptic digest, and one missed cleavage. All analyses included dynamic oxidation of methionine (+15.9949 m/z) as well as static alkylation of cysteine (+57.0215 m/z; iodoacetamide alkylation). Filter parameters were Xcorr scores (+1 ion) 1.5, (+2 ion) 2.0, (+3 ion) 2.5, deltaCn 0.08, and peptide probability >0.05. Residues photolabed by AziP*m* were identified by searching protein peptides for an additional 216.076 Da, which corresponds to the predicted mass of the AziP*m* adduct. Both the in-solution and in-gel sequential trypsin/chymotrypsin digests were searched without enzyme specification with a false discovery rate of 0.01. Samples were conducted in triplicate and samples containing no photoaffinity ligand were treated similarly to control for false positive detection of photoaffinity ligand modifications. To confirm the photolabeled adduct, mass spectrometry work was repeated at the Proteomics Core Facility of the Wistar Institute (Philadelphia, PA, USA).

**Molecular dynamics simulations.** We used a structural model determined by cryo-EM, purified from porcine muscle (2). Only the central pore domain of RyR1 – which itself is a functional channel (10) – was simulated, for the sake of computational feasibility. A system containing the entire protein would be prohibitively large. Simulating the whole protein to a timescale where significant global conformational changes could be observed, especially as part of an allosteric mechanism that would influence pore opening from distant binding sites, was computationally impractical, particularly for demanding free energy perturbation MD simulations.

The molecular mechanics parameters for propofol were taken from a previous simulation study of propofol (11). All simulations were conducted with NAMD 2.14 or 3 (12), with GPUs used for equilibrium MD simulations. The CHARMM36 force field (13,14) and TIP3P water model were used. Systems were constructed using CHARMM-GUI (15,16). Production simulations were conducted in the isothermic-isobaric ensemble with Langevin thermostat. The lipid bilayers consisted of 70% 1-palmitoyl-2-oleoyl-sn-glycero-3-phosphocholine (POPC) and 30% cholesterol.

We calculated the absolute binding free energy of propofol ${\Delta G}_{bind}^{\circ}$. Free energy perturbation (FEP) molecular dynamics simulations were conducted according to the Streamlined Alchemical Free Energy Perturbation (SAFEP) methodology, described in detail in (17). We followed the set-up and analysis procedure described in (18). Briefly, SAFEP uses a limited set of restraints on the ligand to maintain its bound conformation during alchemical transformations and improve sampling of states that most contribute to the binding free energy. The restraints are then corrected for to yield an accurate absolute binding free energy. The overall expression is

$${\Delta G}_{bind}^{\circ}={-\Delta G}_{site}+{\Delta G}_{DBC}-{\Delta G}_{V}^{\circ}+{\Delta G}_{bulk}$$

where ${\Delta G}_{bulk}$ is the energy of decoupling the unbound ligand from solvated to gas phase, ${\Delta G}_{V}^{\circ}$ and ${\Delta G}_{DBC}$ energies of volumetric and distance-from-bound-conformation (DBC) restraints respectively, and ${-\Delta G}_{site}$ is the energy of coupling the ligand from gas phase to the protein-bound state. ${\Delta G}_{bulk}$ and ${\Delta G}_{site}$ were calculated using FEP MD, ${\Delta G}_{DBC}$ using thermodynamic integration, and ${\Delta G}_{V}^{\circ}$ parametrically.

For the calculation of ${\Delta G}_{bind}^{\circ}$, 160 windows were used; for ${\Delta G}_{bulk}$, 481 windows; and for ${\Delta G}_{DBC}$, 40 windows. Interleaved double-wide sampling was used. Restraints were imposed using the Colvars module (19). The Bennett Acceptance Ratio method was used to calculate free energy differences in FEP calculations.

**References for Detailed Methods**

1. Cholak S, Saville JW, Zhu X, Berezuk AM, Tuttle KS, Haji-Ghassemi O, et al. Allosteric modulation of ryanodine receptor RyR1 by nucleotide derivatives. Structure. 2023 Jul 6;31(7):790-800.e4.

2. Woll KA, Haji-Ghassemi O, Van Petegem F. Pathological conformations of disease mutant Ryanodine Receptors revealed by cryo-EM. Nat Commun. 2021 Feb 5;12(1):807.

3. Sawas AH, Pentyala SN, Rebecchi MJ. Binding of volatile anesthetics to serum albumin: measurements of enthalpy and solvent contributions. Biochemistry. 2004 Oct 5;43(39):12675–85.

4. Shomer NH, Mickelson JR, Louis CF. Ion selectivity of porcine skeletal muscle Ca2+ release channels is unaffected by the Arg615 to Cys615 mutation. Biophys J. 1994 Aug;67(2):641–6.

5. Tong J, Oyamada H, Demaurex N, Grinstein S, McCarthy TV, MacLennan DH. Caffeine and Halothane Sensitivity of Intracellular Ca2+ Release Is Altered by 15 Calcium Release Channel (Ryanodine Receptor) Mutations Associated with Malignant Hyperthermia and/or Central Core Disease*. J Biol Chem. 1997 Oct 17;272(42):26332–9.

6. Volpatti JR, Endo Y, Knox J, Groom L, Brennan S, Noche R, et al. Identification of drug modifiers for RYR1-related myopathy using a multi-species discovery pipeline. Stainier DY, Mumm JS, Mumm JS, Pessah I, Benian GM, Henry C, editors. eLife. 2020 Mar 30;9:e52946.

7. Fellner SK, Arendshorst WJ. Ryanodine receptor and capacitative Ca2+ entry in fresh preglomerular vascular smooth muscle cells. Kidney Int. 2000 Oct 1;58(4):1686–94.

8. Woll KAB, Dailey WP, Brannigan G, Eckenhoff RG. Shedding Light on Anesthetic Mechanisms: Application of Photoaffinity Ligands. Anesth Analg.

9. Cox J, Mann M. MaxQuant enables high peptide identification rates, individualized p.p.b.-range mass accuracies and proteome-wide protein quantification. Nat Biotechnol. 2008 Dec;26(12):1367–72.

10. Bai XC, Yan Z, Wu J, Li Z, Yan N. The Central domain of RyR1 is the transducer for long-range allosteric gating of channel opening. Cell Res. 2016 Sep;26(9):995–1006.

11. LeBard DN, Hénin J, Eckenhoff RG, Klein ML, Brannigan G. General Anesthetics Predicted to Block the GLIC Pore with Micromolar Affinity. PLoS Comput Biol. 2012 May 31;8(5):e1002532.

12. Phillips JC, Braun R, Wang W, Gumbart J, Tajkhorshid E, Villa E, et al. Scalable molecular dynamics with NAMD. J Comput Chem. 2005 Dec;26(16):1781–802.

13. Best RB, Zhu X, Shim J, Lopes PEM, Mittal J, Feig M, et al. Optimization of the Additive CHARMM All-Atom Protein Force Field Targeting Improved Sampling of the Backbone ϕ, ψ and Side-Chain χ1 and χ2 Dihedral Angles. J Chem Theory Comput. 2012 Sep 11;8(9):3257–73.

14. Klauda JB, Venable RM, Freites JA, O’Connor JW, Tobias DJ, Mondragon-Ramirez C, et al. Update of the CHARMM All-Atom Additive Force Field for Lipids: Validation on Six Lipid Types. J Phys Chem B. 2010 Jun 17;114(23):7830–43.

15. Jo S, Kim T, Iyer VG, Im W. CHARMM-GUI: A web-based graphical user interface for CHARMM. J Comput Chem. 2008 Aug 1;29(11):1859–65.

16. Jo S, Lim JB, Klauda JB, Im W. CHARMM-GUI Membrane Builder for Mixed Bilayers and Its Application to Yeast Membranes. Biophys J. 2009 Aug 7;97(1):50–8.

17. Salari R, Joseph T, Lohia R, Hénin J, Brannigan G. A Streamlined, General Approach for Computing Ligand Binding Free Energies and Its Application to GPCR-Bound Cholesterol. J Chem Theory Comput. 2018 Nov 13;

18. Santiago-McRae E, Ebrahimi M, Sandberg JW, Brannigan G, Hénin J. Computing Absolute Binding Affinities by Streamlined Alchemical Free Energy Perturbation (SAFEP) [Article v1.0]. Living J Comput Mol Sci. 2023 Oct 23;5(1):2067–2067.

19. Fiorin G, Klein ML, Hénin J. Using collective variables to drive molecular dynamics simulations. Mol Phys. 2013 Dec 1;111(22–23):3345–62.
